# The Cytoprotective Enzyme Heme Oxygenase-1 Suppresses Pseudorabies Virus Replication *in vitro*

**DOI:** 10.3389/fmicb.2020.00412

**Published:** 2020-03-13

**Authors:** Angke Zhang, Bo Wan, Dawei Jiang, Yanan Wu, Pengchao Ji, Yongkun Du, Gaiping Zhang

**Affiliations:** College of Animal Sciences and Veterinary Medicine, Henan Agricultural University, Zhengzhou, China

**Keywords:** heme oxygenase-1, carbon monoxide, biliverdin, antiviral effect, pseudorabies virus

## Abstract

Pseudorabies virus (PRV) infection brings about great economic losses to the swine industry worldwide, as there are currently no effective therapeutic agents or vaccines against this disease, and mutations in endemic wild virulent PRV strains result in immune failure of traditional vaccines. Heme oxygenase-1 (HO-1) catalyzes the conversion of heme into biliverdin (BV), iron and carbon monoxide (CO), all of which have been demonstrated to protect cells from various stressors. However, the role of HO-1 in PRV replication remains unknown. Thus, the present study aimed to investigate the effect of HO-1 on PRV replication and determine its underlying molecular mechanisms. The results demonstrated that induction of HO-1 via cobalt-protoporphyrin (CoPP) markedly suppressed PRV replication, while HO-1 specific small interfering RNA or inhibitor zinc-protoporphyrin partially reversed the inhibitory effect of CoPP on PRV replication. Furthermore, overexpression of HO-1 notably inhibited PRV replication, while knockdown of endogenous HO-1 expression promoted PRV replication. Mechanism analyses indicated that the HO-1 downstream metabolites, CO and BV/BR partially mediated the virus suppressive effect of HO-1. Taken together, the results of the present study suggest that HO-1 may be developed as a novel endogenous antiviral factor against PRV, and the HO-1/BV/CO system may constitute a unique antiviral protection network during PRV infection and interaction with host cells.

## Introduction

Pseudorabies (PR), caused by the pseudorabies virus (PRV) and characterized by severe reproductive, respiratory and neurological disorders, is one of the most devastating infectious diseases in pigs that brings about great economic losses to the swine industry worldwide (Nauwynck et al., 2007; Freuling et al., 2017). PRV belongs to the Herpesviridae family, α-herpesvirinae subfamily and varicellovirus genus (Mettenleiter, 2000). Although the virus has been reported to infect a variety of mammals, including ruminants, carnivores and rodents and cause ∼100% mortality, pigs are the only natural hosts for PRV in which latency is established (Mettenleiter, 1991). Since the 2011 outbreak of PRV in some farms of China, in which pigs were vaccinated with Bartha-K61, subsequent mutations in PRV strains have introduced great challenges in the prevention and control of PR (An et al., 2013; Yu et al., 2014). To date, several precautionary measures have been taken to prevent and control PR in China; however, due to the sophisticated factors associated with the virus biology, transmission and infection of PRV remains difficult to completely control.

Heme oxygenase-1 (HO-1) is ubiquitously expressed in mammalian cells and catalyzes the conversion of heme into carbon monoxide (CO), iron and biliverdin (BV), which is subsequently converted into bilirubin (BR) via NADPH-dependent biliverdin reductase (BVR) (Tenhunen et al., 1969). HO-1 is considered to exert its cytoprotective effect via the antioxidant, antiapoptotic and anti-inflammatory activities of its downstream metabolites (Ryter and Choi, 2009, 2016). Previous studies have reported that HO-1 exerts a protective role against oxidative stress (Lee J.C. et al., 2014). Virus infection of host cells is often accompanied by oxidative stress stimulation, which means the potential role of HO-1 during virus infection has recently been gaining recognition. Thus far, a number of studies have demonstrated the antiviral activity of HO-1. For example, induction or overexpression of HO-1 has been reported to inhibit infection of influenza (Hashiba et al., 2001), human immunodeficiency virus (HIV) (Devadas and Dhawan, 2006), hepatitis B virus (HBV) (Protzer et al., 2007), dengue virus (DENV) (Tseng et al., 2016), and human respiratory syncytial virus (RSV) (Espinoza et al., 2017). HO-1 has also exhibited antiviral properties within viruses effecting domestic animals. For instance, HO-1 can suppress the replication of bovine viral diarrhea virus (BVDV) *in vitro* (Xiao et al., 2014). These previous studies emphasize the potential use of this cytoprotective enzyme as a virucidal agent. However, the molecular mechanism underlying the antiviral effect of HO-1 still remains largely unknown.

BV and BVR are well characterized signaling cascades and the only metabolic pathway producing BR (Maines, 2005; Kapitulnik and Maines, 2009). The conversion of BV to BR via BVR is a physiological process, synchronized with the metabolism of heme (Greenberg, 2002; Bach, 2006). Previous studies have demonstrated that the BV/BVR system displays effective anti-inflammatory and antiviral activities (Sass et al., 2004; Wegiel et al., 2011). For example, as a downstream metabolite of HO-1, BV has been demonstrated to act as a key effector against HCV replication by activating the antiviral IFN response and inhibiting the NS3/4A protease activity of HCV (Lehmann et al., 2010; Zhu et al., 2010). HO-1 derived from BR has been reported to suppress human herpes simplex type 1 virus (HSV-1) and EV71 infection, as well as protease activity of DENV and HIV (Santangelo et al., 2012; Olagnier et al., 2014; Liu et al., 2016). Iron ions derived from HO-1 have been demonstrated to participate in key cellular processes that are dependent on this metal, either promoting or suppressing the translation of particular mRNAs, depending on its concentrations (Eisenstein et al., 1991). Elevated intracellular iron concentrations have been implicated in the activation of the cytoprotective NF-κB signaling pathway, which can effectively reverse Fas-mediated cell apoptosis (Choi et al., 2004). Notably, HO-1-derived iron was reported to suppress subgenomic replication of HCV by inactivating its NS5B RNA-dependent RNA polymerase activity (Fillebeen et al., 2005), suggesting its potential antiviral activity.

CO is another metabolite of HO-1, which has been demonstrated to exert anti-inflammatory, antiapoptotic and cytoprotective effects in several types of diseases (Otterbein, 2002; Chung et al., 2008). CO has been reported to inhibit the expression of proinflammatory molecules on the cell surface (Riquelme et al., 2015a), regulation of mitochondrial function (Riquelme et al., 2015b) and inhibit T cell activation (Mackern-Oberti et al., 2015). With relevance to the present study, previous findings have reported an association between CO and viral replication in host cells. For example, CO has been demonstrated to suppress ROS generation in EV71-infected cells, thus restraining viral replication in host cells (Tung et al., 2011). Another study reported that HO-1-derived CO suppresses the NF-κB signaling pathway, while activating the cGMP/PKG cascade to impede porcine reproductive and respiratory syndrome virus (PRRSV) replication in its permissive cells (Zhang et al., 2017). The discovery of CO-releasing molecules (CORMs) provides pharmacological tool to further determine the bioactive properties of CO (Motterlini et al., 2002).

As mentioned above, the role of HO-1 and its underlying molecular mechanisms during PRV infection remain unclear. Given that the HO-1 products, BV and CO, play key roles in mediating the cytoprotective function of this molecule that exerts antiviral effects, studying the role of this enzyme in relation to PRV replication may help identify novel therapeutic approaches to overcome viral infection. The present study aimed to investigate the role of HO-1 activity on the infection and replication of PRV in porcine kidney (PK)-15 cells and swine testis (ST) cells, and determine the potential molecular mechanisms involved in this process. The results demonstrated for the first time that induction or overexpression of HO-1 markedly inhibits PRV replication, while knockdown of endogenous HO-1 expression facilitates PRV replication, indicating that HO-1 may act as an effective endogenous antiviral factor. Further mechanistic studies revealed that the downstream metabolites of HO-1 (BV/BR and CO) partially mediate the antiviral activity of this enzyme. Taken together, the results of the present study suggest that HO-1 and its products (BV and CO) may function in suppressing PRV replication, thus act as novel therapeutic targets against PRV infection.

## Materials and Methods

### Cells and Virus Strain

Vero cells derived from African green monkey kidney cells and PK-15 cells were both obtained from China Center for Type Culture Collection (CCTCC) and were maintained in Dulbecco’s Modified Eagle Medium (DMEM) supplemented with 10% fetal bovine serum (FBS), 100 IU/ml penicillin and 0.1 mg/ml streptomycin (Thermo Fisher Scientific, Inc.) at 37°C in 5% CO_2_. ST cells (obtained from CCTCC) were maintained in 10% FBS + MEM (Gibco; Thermo Fisher Scientific, Inc.), followed by the aforementioned culture conditions. The Bartha-K61 PRV strain (GenBank ID: JF797217.1) was kindly provided by Professor Jiang Wang of Henan Agricultural University.

### Antibodies and Other Reagents

Rabbit polyclonal antibody directly against PRV glycoprotein B (gB) and mouse anti-HO-1 monoclonal antibody were both purchased from Abcam. Mouse anti-α-tubulin monoclonal antibody was obtained from Sigma-Aldrich; while rabbit anti-BVR monoclonal antibody was purchased from Cell Signaling Technology, Inc.

Cobalt-protoporphyrin (CoPP), a long-established HO-1 inducer; zinc-protoporphyrin (ZnPP), a HO-1 inhibitor; CO-releasing molecule-2 (CORM-2), a transition metal carbonyl that can liberate CO to elicit direct biological activities and BV were all purchased from Sigma-Aldrich; while CO scavenger hemoglobin (Hb) was purchased from Beyotime Institute of Biotechnology. Inactive (i)CORM-2 was prepared by incubating CORM-2 dissolved in DMSO for 24 h at 37°C in 5% CO_2_ to release CO. Small interfering (si)BVR and siHO-1, and their corresponding negative controls (NCs) were synthesized by Shanghai GenePharma Co., Ltd.

### Modulation of HO-1 Activity and the Contents of CO and BV in Cells

PK-15 or ST cells were inoculated with PRV at a multiplicity of infection (MOI) of 0.01, in DMEM at 37°C for 1 h. Cells were subsequently washed three times with PBS and cultured in 3% FBS + DMEM supplemented with 0, 10, 25, and 50 μM of CoPP (dissolved in 0.1 M of NaOH) for 36 h at 37°C. CORM-2 (dissolved in DMSO) was used as a CO donor, and the final concentrations were identical to that of CoPP, which was added 1 h post-infection (hpi), while 50 μM of iCORM-2 was used as a control. Exogenous BV (dissolved in 0.1 of M NaOH) was used to assess the effect of BV on PRV infection, and the concentrations were identical to that of CORM-2.

### Reverse Transcription-Quantitative PCR (RT-qPCR)

Following treatment, cells were harvested and total RNA were extracted using RNAiso plus reagent (Takara Biotechnology Co., Ltd.), according to the manufacturer’s protocol. Total RNA (500 ng) was reverse transcribed into cDNA using the PrimeScript^®^ RT reagent kit (Takara Biotechnology Co., Ltd.). qPCR was subsequently performed on a Light Cycler 480 SYBR Green I Master fluorescence quantitative PCR instrument, using FastStart Universal SYBR green master (Roche Diagnostics GmbH). The following primer sequences were used for qPCR: pHO-1 forward, 5′-GGCTGAGAATGCCGAGTT-3′ and reverse, 5′-ATGTAGCGGGTGTA GGCGTGGG-3′; gPRV forward, 5′-GGCGTACTGGCGCACTCTG-3′ and reverse, 5′-ATGTC CCCGACGATGAAGC-3′; gB forward, 5′-CCTCGTCCACG TCGTC CTC-3′ and reverse, 5′-GGCATCGCCAACTTCTTCC-3′; and β-actin forward, 5′-T CCCTGGAGAAGAGCTACGA-3′ and reverse, 5′-AGCACTGTGTTGGCGTA CAG-3′. The following thermocycling conditions were used for qPCR: Initial denaturation at 95°C for 10 min; 40 cycles at 95°C for 20 s, 55°C for 30 s and 72°C for 20 s. Relative mRNA levels were quantified using the 2^–ΔΔ*Ct*^ method and normalized to the internal reference gene β-actin.

Regarding detection of supernatant virus copies, the PRV gB segment was cloned into the pEASY-T1 vector (TransGen Biotechnology Co., Ltd.), in order to construct the pEASY-T1-gB recombinant plasmid and generate a standard curve. The standard curve was plotted from the results of parallel PCRs performed on serial dilutions of standard DNA, while absolute quantities of supernatant RNA were calculated by normalization to the standard curve.

### siRNA Treatment

siRNAs targeting the swine HO-1 coding sequences (GenBank ID: NM_001004027.1) were synthesized by Shanghai GenePharma Co., Ltd., and scrambled sequences were used as the non-targeted siRNA control. The HO-1 siRNA sequences were as follows: Sense, 5′-CGUCCUUGUACCACAUCUAdTdT-3′ and anti-sense, 5′-UAGAUGUGGUACAAGGACGdTdT-3′; and BVR sense, 5′-UCCUC AGCGUUCCUGAACCUGTT-3′ and BVR anti-sense, 5′-CAGGUUCAGGAACGC UGAGGATT-3′. siRNAs were transfected into PK-15 cells at a final concentration of 100 nM, using the X-tremeGENE HP siRNA transfection reagent (Roche Applied Science) and incubated for 12 h at 37°C. Subsequently, cells were infected with 0.01 MOI of PRV prior to treatment with 50 μM of CoPP or BV, from 1 hpi onwards. HO-1 or BVR knockdown efficiency was assessed via western blotting, and the effect of HO-1 or BVR knockdown on PRV infection was determined as described for the supernatants virus copies and PRV gB expression detection.

### Cell Viability and Cytotoxicity Assay

Cell viability and cytotoxicity were assessed via Cell Counting Kit-8 (CCK-8; Beyotime Institute of Biotechnology), according to the manufacturer’s protocol. Briefly, PK-15 or ST cells were seeded into 96-well plates at a density of 1 × 10^4^ cells/well. Cells were cultured with 10% FBS + DMEM for 24 h at 37°C in 5% CO_2_, prior to replacing the culture medium with 3% FBS + DMEM supplemented with varying concentrations of CoPP, BV or CORM-2 and incubated for 36 h at 37°C. CCK-8 reagent (10 μl) was added to each well containing 100 μl of DMEM and incubated for 2 h at 37°C, and cell viability was subsequently analyzed at a wavelength of 450 nm, using an epoch microplate spectrophotometer (BioTek Instruments, Inc.). The 50% cytotoxic concentration (CC_50_), defined as the concentration that causes visible changes in 50% of intact cells, was determined by comparing the CoPP-treated group with the untreated group, using GraphPad Prism software (version 5.0; GraphPad Software, Inc.). The maximal non-cytotoxic concentration (MNCC) was defined as the maximal concentration of CoPP that did not exhibit toxic effects via CCK-8 analysis.

### Cytopathic Effect (CPE) Inhibition Assay

The antiviral activity of CoPP against PRV was determined via the CPE inhibition assay. Briefly, PK-15 or ST cells were seeded into 96-well plates at a density of 1 × 10^4^ cells/well about 24 h before infecting with equal volumes of PRV (0.01 MOI), following by the addition of twofold serial dilutions of CoPP to each well. The MNCC was set as the highest concentration for this experiment. Following cell culture for 72 h at 37°C, the CPE was observed in each well. PRV-infected PK-15 or ST cells without CoPP treatment were used as the control. The CoPP concentration decreasing CPE by 50%, compared with the control group, was estimated using GraphPad Prism software and defined as the half maximal inhibitory concentration (IC_50_), expressed in μmol/l. The selectivity index (SI) was calculated from the ratio of CC_50_ to IC_50_.

### Indirect Immunofluorescence Assay (IFA)

PK-15 or ST cells were seeded into 24-well plates at a density of 5x10^4^ cells/well, and then infected with 0.01 MOI of PRV for 24 h at 37°C, in the presence or absence of 10, 25, and 50 μM of CoPP. The infected cells were fixed using 4% paraformaldehyde for 30 min at room temperature, washed three times in PBS and permeabilized with 0.5% Triton X-100 for 30 min at room temperature. The fixed cells were subsequently blocked with 1% bovine serum albumin (BSA; Beyotime Institute of Biotechnology) for 1 h at room temperature and incubated with rabbit anti-PRV gB or mouse anti-HO-1 antibodies (1:300; Abcam), diluted in 1% BSA supplemented with PBS, for 1 h at room temperature. Cells were washed three times with PBS, prior to incubation with goat anti-mouse IgG H&L (Alexa Fluor 594) or goat anti-rabbit IgG H&L (Alexa Fluor 488) (both 1:300; Abcam), diluted in 1% BSA supplemented with PBS, for 1 h at room temperature. Cell nuclei were counterstained with DAPI (Beyotime Institute of Biotechnology) for 5 min at room temperature. Samples were washed three times with PBS and the positively stained cells were observed under a confocal microscope with a magnification of 100 x (Leica Microsystems GmbH).

### ZnPP Treatment of Target Cells

In order to determine whether HO-1 enzyme activity was essential for its antiviral function, PK-15 or ST cells with 80% confluence were treated with 0, 20, 40, and 60 μM of ZnPP for 12 h at 37°C. Cells were washed three times with PBS and subsequently infected with 0.01 MOI of PRV for 1 h at 37°C. The viral suspension was replaced with 3% FBS + DMEM containing 50 μM of CoPP. Cells were harvested to determine gB expression via western blotting, while supernatants virus copies were detected via RT-qPCR analysis, at 36 hpi. PK-15 or ST cells with 80% confluence were treated with 0, 20, 40, and 60 μM of ZnPP for 24 h at 37°C, in order to investigate the effect of ZnPP on HO-1 protein expression.

### Measurement of Intracellular Oxidative Stress

Intracellular reactive oxygen species (ROS) and reactive nitrogen species (RNS) production in PK-15 cells before and after PRV infection was assessed using DCFH-DA and DAF-FM DA fluorescence probe (Beyotime Institute of Biotechnology), according to the manufacturer’s protocol. PK-15 cells were seeded into 6-well plates at a density of 2 × 10^5^ cells/well 24 h prior to exposure with PRV (MOI of 0.01). At 24 hpi, the virus supernatant was discarded, cells were washed three times with PBS and incubated with DMEM supplemented with 10 μM/l of DCFH-DA probe at 37°C for 20 min. Subsequently, cells were re-washed three times with PBS. Cells were observed under a confocal fluorescence microscope (100 x; Leica Microsystems GmbH) and assessed via flow cytometry analysis.

For analysis of intracellular RNS level, PK-15 cells were plated in 6-well plates at a density of 2 × 10^5^ cells/well 1 day before and followed by infecting with 0.01 MOI of PRV for 24 h. Then the old medium was replaced with fresh serum free DMEM containing 5 μM/l DAF-FM DA probe and inubated for 20 min at 37°C. After washing using PBS for 3 times, cells were subjected to flow cytometry analysis. PK-15 cells not infected with PRV were used as the control in both experiments.

### Generation of Stable Cell Lines

In order to generate cell line stably expressing porcine HO-1, the HO-1 gene was cloned into the lentiviral expression vector, pTRIP-puro to construct pTRIP-pHO-1-puro. Subsequently, the recombinant plasmids (0.9 μg) were co-transfected into 293T cells, with pMDG.2 (1.0 μg) and psPAX2 (1.8 μg) plasmids, using X-tremeGENE HP DNA transfection reagent and Opti-MEM to construct pseudotyped lentiviral vectors. Supernatants containing the pseudotyped lentiviruses were collected at 48 h post-transfection and centrifuged at 12,000 × *g* for 10 min at 4°C to remove cell debris. Subsequently, the supernatants were filtered with 0.22 μM filter and stored at −80°C for subsequent experimentation.

PK-15 or ST cells that reached 80% confluence were transduced with the pseudotyped lentiviruses expressing HO-1 and supplemented with 1 μg/ml of polybrene (Sigma-Aldrich; Merck KGaA). After 24 h, the culture medium was replaced with 10% FBS + DMEM/MEM supplemented with 8 μg/ml of puromycin (Sigma-Aldrich; Merck KGaA), which was changed every 48 h. mRNA and protein expression levels of PK-15 or ST cell with upregulated HO-1 were determined via RT-qPCR and western blotting, respectively.

HO-1 knockdown in PK-15 and ST cell lines was constructed by cloning the short hairpin (sh)RNA sequence of HO-1 into pLKO.1-EGFP-puro expression plasmid. The shRNA sequences targeting HO-1 were as follows: Sense, 5′-CCGGC GTCCTTGTACCACATCTACTCGAGTAGATGTGGTACAAG GACGTTTTTG-3′; and shRNA-control, 5′-GCACTACCA GAGCTAACTCAGATAGTACT-3′. Stable cell lines were established via the aforementioned process and those with HO-1 knockdown were detected via RT-qPCR and western blotting analyses.

### Enzyme-Linked Immunosorbent Assay (ELISA)

In order to determine whether induction of HO-1 or PRV infection leads to abnormal changes of intracellular BV, the amount of BR was initially assessed as a reflection of intracellular BV levels after treatment with CoPP or PRV infection of host cells. PK-15 or ST cells were seeded into 6-well plates at a density of 2 × 10^5^ cells/well. Following cell culture for 24 h at 37°C, the medium was changed to 3% FBS + DMEM, with or without 50 μM of CoPP, or cells were infected with 0.01 MOI of PRV. Cells and supernatants were harvested at 36 h following treatment with CoPP, or at 12, 24, 36, 48 h post-PRV infection, and subsequently frozen and thawed three times. Cell lysates were centrifuged at 12,000 × g for 10 min at 4°C and supernatants were collected for BR detection using a BR ELISA kit (Elabscience Biotechnology Co., Ltd.), according to the manufacturer’s protocol. Samples were subsequently analyzed at a wavelength of 540 nm, using an epoch microplate spectrophotometer.

As Hb has a high affinity for CO (forming HbCO) (Tung et al., 2011), the intracellular CO levels were determined following HO-1 induction or PRV infection using a commercially available HbCO ELISA kit (Elabscience Biotechnology Co., Ltd.), according to the manufacturer’s protocol. Briefly, PK-15 or ST cells were seeded into 6-well plates at a density of 2 × 10^5^ cells/well. After 24 h, cells were treated with 3% FBS + DMEM supplemented with 50 μM of CoPP, with or without 50 μg/ml of Hb, or inoculated with PRV as aforementioned. Cells and supernatants were harvested at 36 h post-treatment or at 12, 24, 36, 48 h post-PRV infection to detect HbCO via ELISA. Samples were subsequently analyzed at a wavelength of 450 nm, using an epoch microplate spectrophotometer.

### Viral Attachment and Entry Assay

PK-15 or ST cells were seeded into 6-well plates at a density of 2 × 10^5^ cells/well 24 h prior to treatment with 0, 10, 25, 50 μM of CoPP for 12 h at 37°C, and followed by washing three times with PBS to remove residual CoPP. Cells were subsequently pre-chilled on ice for 30 min, followed by inoculation with ice-cold PRV (1.0 MOI) for 1 h at 4°C to allow abundant virus adsorption without internalization. Cells were washed extensively with ice-cold PBS to remove unabsorbed viruses and analyzed via RT-qPCR or western blotting.

For the entry assay, PK-15 cells were treated with 0, 10, 25, 50 μM of CoPP for 12 h at 37°C. After washing with PBS for three times, pre-cold cells were inoculated with PRV (1.0 MOI) for 1 h at 4°C to allow viruses to bind without internalizing into cells. Viruses were subsequently discarded and washed three times with ice-cold PBS. Cells were transferred to 37°C for 1 h to promote virus entry into cells. Following extensive washing with PBS, cells were treated with trypsin for 30 s at 37°C and washed three times with PBS to remove the non-internalized virions on the cell surface. Subsequently, cells were harvested for viral genome abundance and gB protein detection via RT-qPCR and western blotting, respectively.

### Western Blotting

Western blotting was performed to determine HO-1 expression and viral proteins under different processing conditions. Treated cells were harvested and lysed on ice for 30 min using NP40 lysis buffer supplemented with protease inhibitor (Beyotime Institute of Biotechnology). Total protein was extracted via centrifugation at 12,000 × g for 10 min at 4°C and quantified using a bicinchoninic acid protein assay kit (Thermo Fisher Scientific, Inc.). A total of 50 μg protein/lane was separated via SDS-PAGE on a 12% gel. The separated proteins were subsequently transferred onto a polyvinylidene difluoride membrane (EMD Millipore) and blocked with 2.5% skim milk (m/v) supplemented with TBST for 1 h at room temperature. The membranes were incubated with primary antibodies against: PRV gB protein (1:1,000), pHO-1 (1:1,000), BVR (1:1,000) and α-tubulin (1:5,000) overnight at 4°C. Membranes were washed three times with TBST. Following the primary incubation, membranes were incubated with HRP-conjugated goat anti-mouse or -rabbit IgG secondary antibodies for 1 h at room temperature. Protein bands were visualized using an ECL chemiluminescent detection system (Pierce; Thermo Fisher Scientific, Inc.), according to the manufacturer’s protocol.

### Statistical Analysis

Statistical analysis was performed using GraphPad Prism software. Data are presented as the mean values ± standard deviation. Differences between two groups was assessed using unpaired Student’s t-test, while one-way analysis of variance (ANOVA) was used to compare differences in three or more groups. *P* < 0.05 was considered to indicate a statistical difference. All experiments were performed in triplicate.

## Results

### PRV Infection Downregulates HO-1 Expression While Stimulates Oxidative Stress Response in Both PK-15 and ST Cells

**FIGURE 1:**
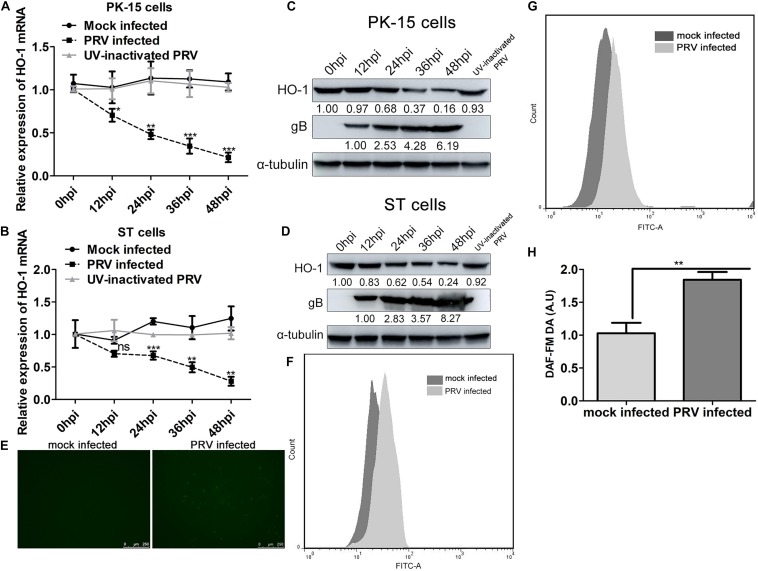
PRV infection suppresses HO-1 expression while stimulates oxidative stress response in both PK-15 and ST cells. **(A,C)** PK-15 or **(B,D)** ST cells were infected with PRV at a MOI of 0.01 for 1 h at 37°C. Cells samples were collected at 0, 12, 24, 36, and 48 hpi to detect HO-1 and PRV gB mRNA and protein levels via RT-qPCR and western blotting, respectively. **(E,F)** PK-15 cells were infected with 0.01 MOI of PRV for 24 h. Then cells were incubated with DMEM supplemented with 10 μM/l of DCFH-DA at 37°C for 20 min. Cells were observed under a confocal fluorescence microscope (100x) and assessed via flow cytometry analysis. **(G,H)** PK-15 cells infected with 0.01 MOI of PRV for 24 h were incubated with DMEM containing 5 μM/l DAF-FM DA and incubated for 20 min at 37°C. After washing using PBS for three times, cells were subjected to flow cytometry analysis. PK-15 cells mock infected with PRV were included as a control in both experiments. Data are presented as the mean ± standard deviation of three independent experiments. *P < 0.05, **P < 0.01, ***P < 0.001. PRV, pseudorabies virus; HO-1, heme oxygenase-1; RT-qPCR, reverse transcription-quantitative PCR.

In order to understand the role of the stress-induced protective enzyme against PRV infection, HO-1 expression was assessed in PK-15 and ST cells during PRV infection. PRV (0.01 MOI) was used to infect PK-15 and ST cells, and mRNA and protein expression levels of HO-1 or gB were analyzed via RT-qPCR and western blotting, respectively. Infection with PRV markedly downregulated HO-1 mRNA expression in both PK-15 and ST cells, as the infection process progressed from 12, 24, 36, and 48 hpi compared with the 0 hpi (Figures 1A,B). In accordance with RT-qPCR analysis, western blotting demonstrated decreased HO-1 protein expression in both PK-15 and ST cells at 12, 24, 36, and 48 hpi compared with the 0 hpi (Figures 1C,D). Furthermore, downregulated HO-1 expression was indicated to be dependent of PRV replication, as UV-inactivated PRV failed to affect HO-1 expression compared with the 0 hpi group (Figures 1A–D). Virus infection of host cells was often associated with oxidative stress stimulation. Thus we determined whether PRV infection affected intracellular oxidative stress using DCFH-DA probe for ROS detection and DAF-FM DA probe for RNS detection. Flow cytometry results revealed that PRV infection markedly promoted intracellular ROS (Figures 1E,F) and RNS (Figures 1G,H) levels compared with mock infected cells, indicating that PRV infection can stimulate cell oxidative stress.

### Induction of HO-1 Inhibits PRV Replication *in vitro*

**FIGURE 2:**
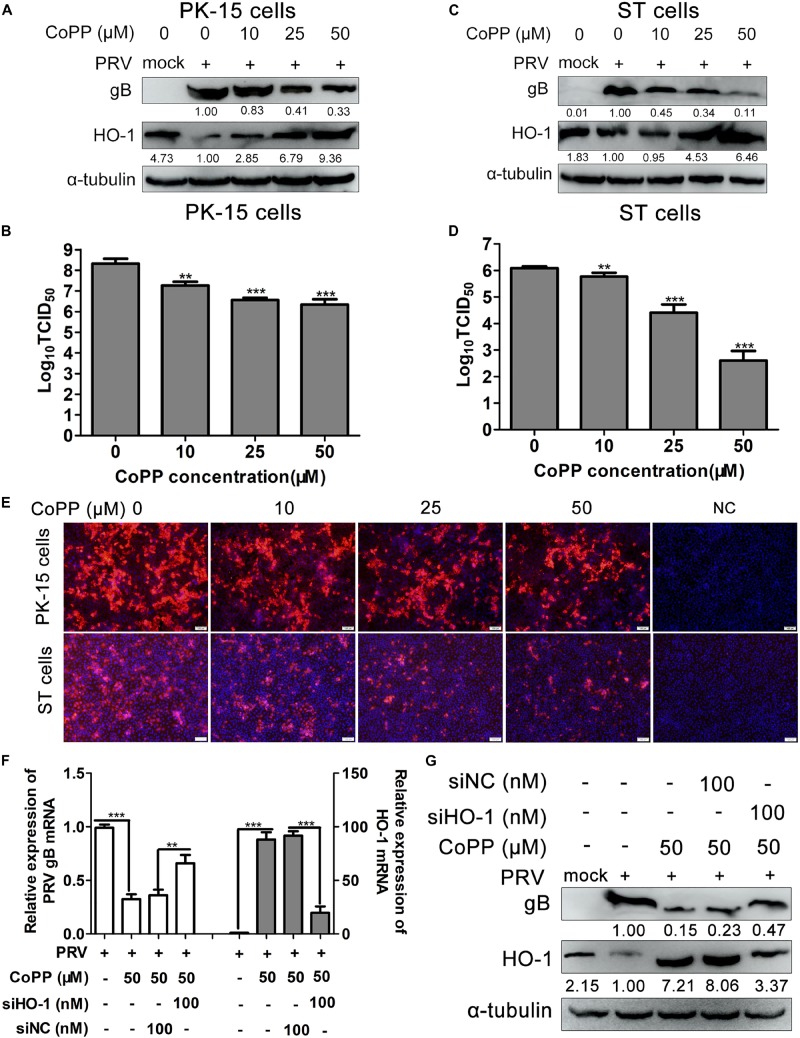
Induction of HO-1 inhibits PRV replication. **(A,B)** PK-15 or **(C,D)** ST cells were inoculated with PRV at a MOI of 0.01 for 1 h at 37°C, followed by treatment with 0, 10, 25, 50 μM of CoPP. Cells and supernatants were harvested to detect HO-1 and gB protein expression via western blotting, and measure supernatant progeny virus titers using TCID_50_, at 36 hpi. **(E)** Indirect immunofluorescence assay was performed. **(F,G)** PK-15 cells were transfected with 100 nM of siHO-1 or siNC for 12 h, then cells were infected with PRV at a MOI of 0.01, in the presence or absence of 50 μM of CoPP. Cells were harvested to detect gB and HO-1 mRNA and protein expression levels via RT-qPCR and western blotting at 36 hpi, respectively. Data are presented as the mean ± standard deviation of three independent experiments. **P < 0.01, ***P < 0.001. HO-1, heme oxygenase-1; PRV, pseudorabies virus; MOI, multiplicity of infection; CoPP, cobalt-protoporphyrin; NC, negative control; RT-qPCR, reverse transcription-quantitative PCR.

Since PRV infection notably aberrated HO-1 expression, the effect of HO-1 on PRV infection and replication was further investigated. First, PK-15 and ST cells were treated with 0, 10, 25, 50 μM of CoPP to evaluate its effect on HO-1 expression. As expected, treatment with CoPP significantly induced HO-1 mRNA and protein expression levels in a dose-dependent manner, in both PK-15 and ST cells (Supplementary Figures 1A–D). In order to investigate the effect of HO-1 on PRV infection and replication, PRV-infected PK-15 and ST cells were treated with 0, 10, 25, 50 μM of CoPP, and PRV infection and replication were subsequently analyzed via western blotting and by assessing the median tissue culture infectious dose (TCID_50_). CoPP increased HO-1 protein expression, while decreasing PRV gB protein expression in both PK-15 and ST cells, in a dose-dependent manner (Figures 2A,C). Furthermore, CoPP decreased supernatants progeny virus titers in a dose-dependent manner (Figures 2B,D). In order to confirm the antiviral activity of HO-1 on PRV infection, IFA was performed to assess PRV gB protein expression, in the presence or absence of 10, 25, and 50 μM of CoPP. IFA analysis demonstrated that induction of HO-1 via CoPP markedly inhibited PRV gB expression (Figure 2E). Taken together, these results suggest that HO-1 may play a key role in suppressing PRV replication *in vitro*.

Subsequently, PK-15 cells were transfected with siHO-1 or siNC, followed by infection with PRV and treatment with 50 μM of CoPP, in order to confirm whether the antiviral activity of CoPP was specifically mediated by HO-1. Samples were harvested and the expression levels of PRV gB and HO-1 were determined. Knockdown of HO-1 partially reversed decreased PRV gB mRNA expression (Figure 2F), as well as the protein (Figure 2G) caused by CoPP treatment compared with the siNC transfection group. These results indicate that HO-1 specifically mediates the antiviral effect of CoPP.

### Enzyme Activity of HO-1 Is Necessary for Its Antiviral Function

**FIGURE 3:**
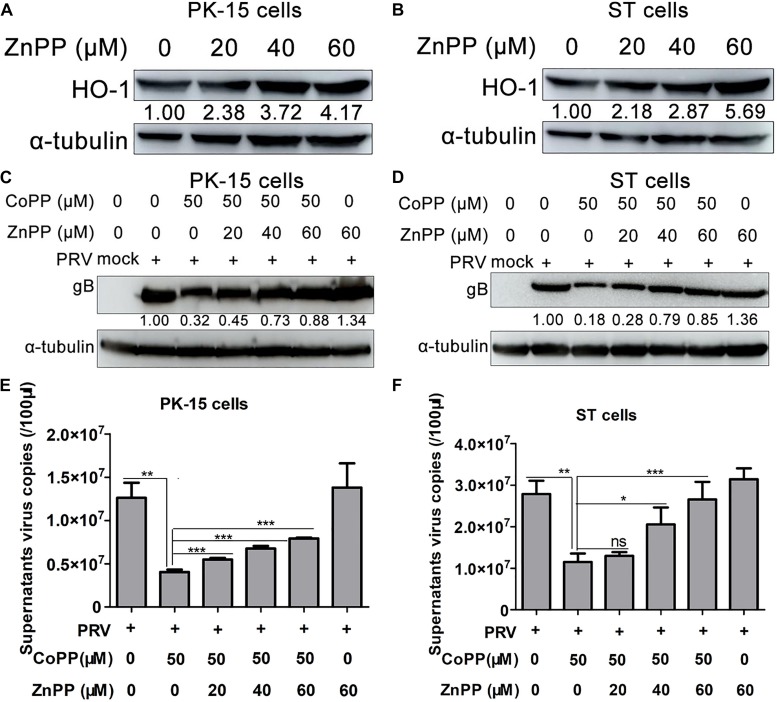
HO-1 specific inhibitor ZnPP partially reverses the inhibitory effect of CoPP on PRV. **(A)** PK-15 or **(B)** ST cells were treated with 0, 20, 40, 60 μM of ZnPP for 24 h. Cells were harvested to detect HO-1 protein expression via western blotting. PK-15 or ST cells were treated with 0, 20, 40, and 60 μM of ZnPP for 12 h at 37°C. Then cells were infected with 0.01 MOI of PRV and followed by incubating with 50 μM of CoPP from 1 hpi onwards. **(C,D)** Cells were collected to detect PRV gB protein expression via western blotting, while **(E,F)** supernatant progeny copies were assessed via RT-qPCR, at 36 hpi. Data are presented as the mean ± standard deviation of three independent experiments. *P < 0.05, **P < 0.01, ***P < 0.001. HO-1, heme oxygenase-1; ZnPP, zinc-protoporphyrin; CoPP, cobalt-protoporphyrin; PRV, pseudorabies virus; RT-qPCR, reverse transcription-quantitative PCR.

ZnPP, a phyrin that upregulates HO-1 protein expression, while inhibiting HO-1 enzymatic activity was used to assess whether it can attenuate PRV inhibition caused by CoPP, in order to determine whether HO-1 enzymatic activity is necessary for CoPP-mediated attenuation of PRV replication. PK-15 or ST cells were treated with 0, 20, 40, 60 μM of ZnPP for 12 h, subsequently cells were harvested and HO-1 expression was determined. The results demonstrated that treatment with ZnPP induced HO-1 protein expression in both cell lines, in a dose-dependent manner (Figures 3A,B). Subsequently, PK-15 or ST cells were treated with varying concentrations of ZnPP for 12 h, followed by infection with PRV and treatment with 50 μM of CoPP for 24 h. As expected, pretreatment with ZnPP partially reversed the inhibitory effect of CoPP on PRV gB expression (Figures 3C,D) and increased supernatants virus copies compared with the CoPP treatment group (Figures 3E,F). Treatment with ZnPP alone slightly induced PRV gB expression, as well as supernatant virus copies (Figures 3C–F). Taken together, these results suggest that HO-1 enzymatic activity is essential for this antiviral effect, and endogenous HO-1 plays a role in restricting virus replication as well.

### Overexpression of HO-1 Suppresses PRV Replication

**FIGURE 4:**
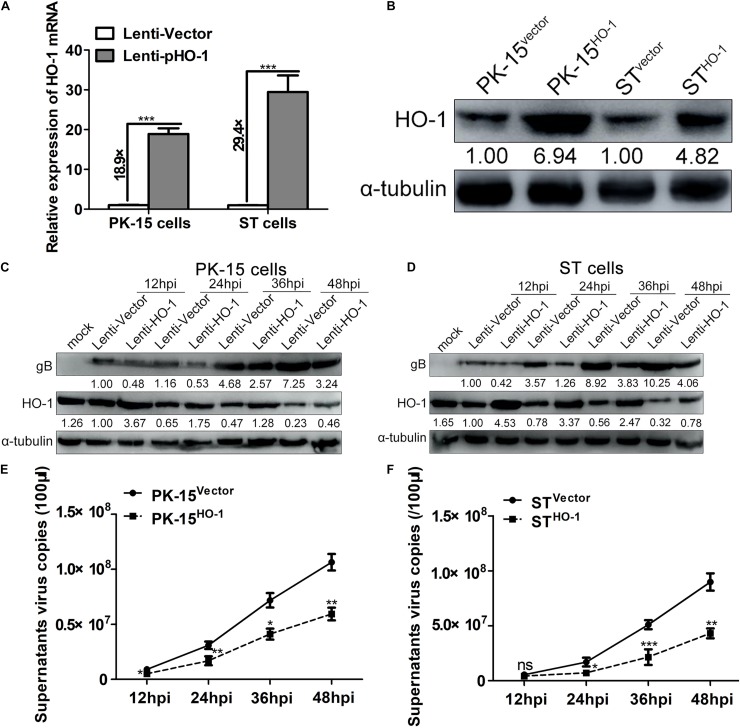
Overexpression of HO-1 suppresses PRV replication. PK-15 or ST cells stably expressing HO-1 were verified via **(A)** RT-qPCR and **(B)** western blotting. PK-15^HO–1^ or ST^HO–1^ cells were infected with PRV at a MOI of 0.01. Samples were harvested at 12, 24, 36, 48 hpi. **(C,D)** Western blotting was performed to assess PRV gB and HO-1 protein expression levels, while **(E,F)** RT-qPCR was performed to detect supernatant progeny virus copies. Data are presented as the mean ± standard deviation of three independent experiments. *P < 0.05, **P < 0.01, ***P < 0.001. HO-1, heme oxygenase-1; PRV, pseudorabies virus; RT-qPCR, reverse transcription-quantitative PCR; MOI, multiplicity of infection.

A recombinant lentivirus overexpressing HO-1 was used to establish cell lines in both PK-15 and ST cells to further verify the effect of HO-1 on PRV replication. First, stable cell lines overexpressing HO-1 were validated, whereby both RT-qPCR and western blotting analyses demonstrated that HO-1 mRNA and protein expression levels were notably upregulated in recombinant cell lines (PK-15^vector^, PK-15^HO–1^, ST^vector^ and ST^HO–1^) compared with the empty vector control group, in both cell types (Figures 4A,B). Subsequently, viral infection characteristics of all cell lines were evaluated at different time points via western blotting and RT-qPCR. Overexpression of HO-1 markedly aberrated gB protein expression and decreased supernatant virus copies compared with the control group at each assessed time point (Figures 4C–F). Furthermore, IFA analysis indicated that overexpression of HO-1 notably abrogated PRV gB expression, while the control groups exhibited apparent more viral infection in both PK-15 and ST cells (Supplementary Figure 2). Taken together, these results suggest that overexpression of HO-1 suppresses PRV replication *in vitro*.

### Knockdown of Endogenous HO-1 Expression Promotes PRV Replication

**FIGURE 5:**
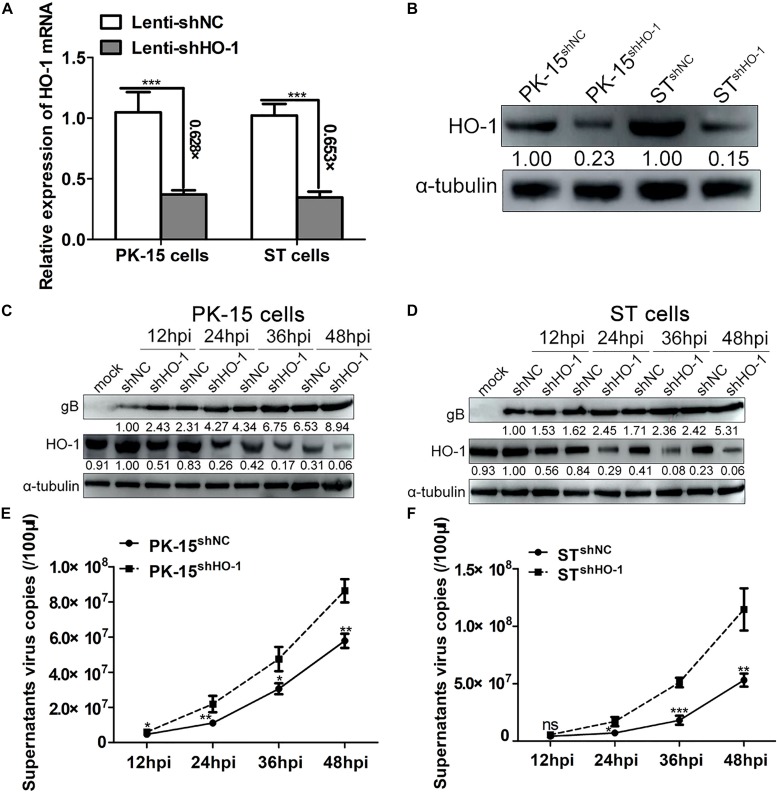
Knockdown of endogenous HO-1 promotes PRV replication. PK-15^shHO–1^ or ST^shHO–1^ cells were identified via **(A)** RT-qPCR and **(B)** western blotting. PK-15^shNC^, PK-15^shHO–1^, ST^shNC^, and ST^shHO–1^ cells were incubated with PRV at a MOI of 0.01. Cells and supernatants were collected at 12, 24, 36, 48 hpi to assess **(C,D)** PRV gB and HO-1 protein expression levels via western blotting, while **(E,F)** supernatant virus copies were detected via RT-qPCR **(E,F)**. Data are presented as the mean ± standard deviation of three independent experiments. *P < 0.05, **P < 0.01, ***P < 0.001. HO-1, heme oxygenase-1; PRV, pseudorabies virus; sh, short hairpin; NC, negative control; RT-qPCR, reverse transcription-quantitative PCR; MOI, multiplicity of infection.

Since upregulation of HO-1 suppressed PRV infection and replication, the association between endogenous basal HO-1 and PRV replication was subsequently investigated. First, PK-15 and ST recombinant cell lines with downregulated HO-1 expression were identified. RT-qPCR analysis demonstrated that HO-1 mRNA expression was markedly downregulated in both PK-15^shHO–1^ (decreased by 62.8%) and ST^shHO–1^ (decreased by 65.3%) cells compared with the control cells (Figure 5A), while western blot analysis indicated downregulated HO-1 protein expression (Figure 5B). PK-15^shNC^, PK-15^shHO–1^, ST^shNC^, or ST^shHO–1^ cells were incubated with PRV (0.01 MOI) and the viral replication kinetics was analyzed. The results demonstrated that shHO-1 markedly aberrated basal level HO-1 protein expression, while simultaneously enhancing PRV gB expression in both PK-15 (Figure 5C) and ST cells (Figure 5D), at 12, 24, 36, and 48 hpi. Furthermore, knockdown of basal level HO-1 expression increased the supernatant progeny virus copies compared with PK-15^shNC^ and ST^shNC^ cells (Figures 5E,F), suggesting that endogenous HO-1 plays an antiviral role during the infection and replication of PRV *in vitro*.

### CoPP Treatment Decreases Viral Infectivity, Without Inducing Cytotoxicity or Interfering With Virus Binding and Entry Into Cells

**FIGURE 6:**
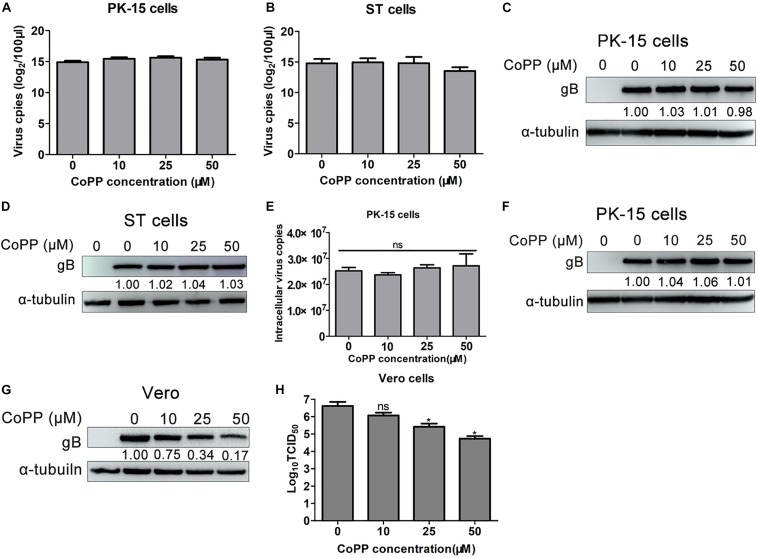
CoPP directly inactivates PRV particles without affecting viral attachment or entry into cells. PK-15 or ST cells treated with 0, 10, 25, 50 μM of CoPP for 12 h at 37°C. After washing three times with PBS to remove residual CoPP, cells were subsequently pre-chilled on ice for 30 min, followed by inoculation with ice-cold PRV (1.0 MOI) for 1 h at 4°C. Cells were washed extensively with ice-cold PBS to remove unabsorbed viruses and analyzed for the virus genome via RT-qPCR **(A,B)** and for the gB protein via western blotting **(C,D)**. For entry assay, following adsorption with PRV (1.0 MOI) for 1 h on ice, CoPP-treated PK-15 cells were transferred to at 37°C. After 1 h, cells were washed with trypsin to remove virions adsorbed on the cell surface, and subsequently harvested to assess virus genome via RT-qPCR **(E)** and gB protein expression via western blotting **(F)**. **(G,H)** PRV virus suspension (0.01 MOI) was co-incubated with CoPP at 37°C for 1 h and used to inoculate Vero cells, and part of the supernatant is directly used to determine the virus titer. After 24 h, cells were harvested for viral replication analysis, and virus titer was tested using TCID_50_. Data are presented as the mean ± standard deviation of three independent experiments. *P < 0.05, **P < 0.01, ***P < 0.001. CoPP, cobalt-protoporphyrin; PRV, pseudorabies virus; MOI, multiplicity of infection; RT-qPCR, reverse transcription-quantitative PCR; gB, glycoprotein B.

To understand the molecular mechanism underlying HO-1 activity of viral protein expression and PRV replication in CoPP-treated cells, a virus-binding assay was performed as previously described by Cheshenko et al. (2013), in order to determine whether equal amounts of virus bound to the surface of CoPP-treated and -untreated PK-15 and ST cells. RT-qPCR analysis indicated that treatment with CoPP did not affect the virus genome content compared with the untreated group, in both PK-15 and ST cells (Figures 6A,B). Western blotting analysis of gB expression demonstrated that equal amounts of virus bound to the surface of untreated and CoPP-treated PK-15 and ST cells, respectively (Figures 6C,D), suggesting that treatment with CoPP does not interfere with virus binding to these cells.

In order to determine how upregulated HO-1, with CoPP prohibited PRV replication, PK-15 cells were inoculated with PRV (0.01 MOI) for 1 h at 4°C to allow viruses to bind without internalizing into cells. Viruses were subsequently discarded and washed three times with PBS. Cells were treated with DMEM containing 0, 10, 25, 50 μM of CoPP for 1 h at 37°C. Subsequently, cells were washed and treated with trypsin prior to harvesting for RT-qPCR and western blotting analyses. No significant differences were observed in PRV gB expression and virus genome content between CoPP-treated and -untreated cells (Figures 6E,F), indicating that treatment with CoPP does not affect virus entry into host cells.

In order to determine whether CoPP affected PRV virulence via its virucidal effect, virus inocula were incubated with varying concentrations of CoPP for 1 h at 37°C, subsequently added to Vero cells and washed to remove CoPP at 1 hpi. Western blot analysis demonstrated that PRV gB expression gradually decreased in CoPP-treated PRV cells compared with the untreated virus control (Figure 6G). Furthermore, supernatants virus titers indicated a downward trend between virus treated with vehicle and virus treated with CoPP (Figure 6H). To eliminate the possibility that the antiviral activity of CoPP was due to its cytotoxicity effect, the CC_50_ and IC_50_ values of CoPP were determined. The IC_50_ and CC_50_ values of CoPP in PK-15 cells were 31.6 and 99.2 μM, respectively, while the SI (CC_50_/IC_50_) value of CoPP was 3.14. Conversely, the IC_50_ and CC_50_ values of CoPP in ST cells were 51.6 and 212.0 μM, respectively, while the SI value of CoPP was 4.11 (Supplementary Figure 3). However, treatment with CoPP was not associated with complete viral inactivation (Figures 6G,H), suggesting that interference with PRV replication occurs partially attribute to downstream mechanism. Taken together, these results suggest that CoPP partially suppresses PRV replication via its direct virucidal effect instead of its cytotoxicity effect.

### HO-1 Metabolite, CO Mediates the Anti-PRV Activity of HO-1

**FIGURE 7:**
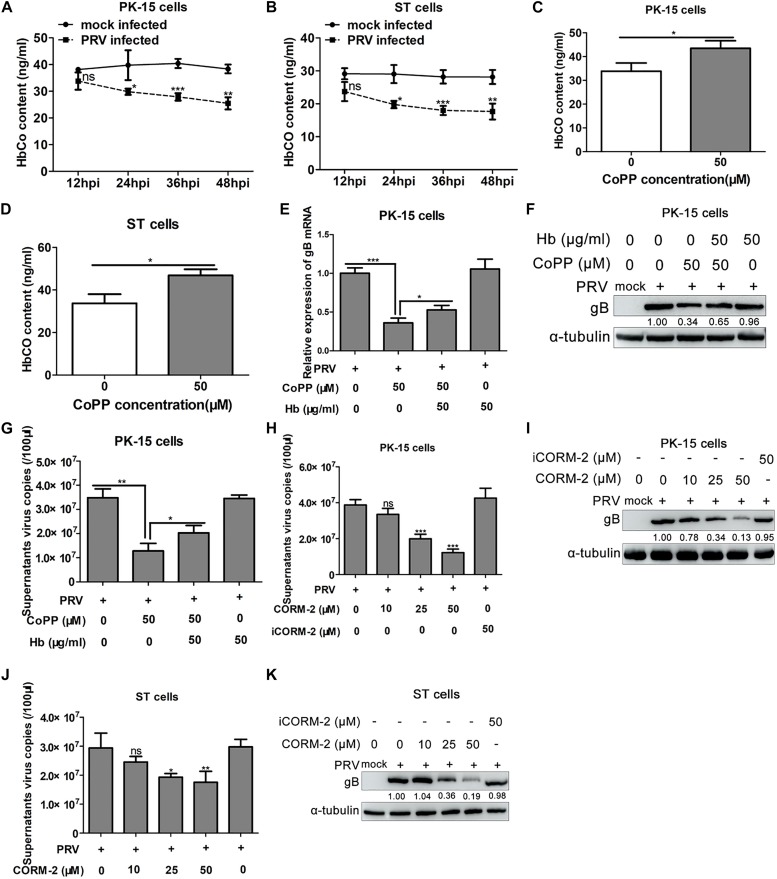
CO mediates the anti-PRV activity of HO-1. **(A)** PK-15 or **(B)** ST cells were infected with PRV at a MOI of 0.01, prior to harvesting at 12, 24, 36, and 48 hpi to detect total HbCO levels via ELISA. **(C)** PK-15 or **(D)** ST cells were incubated with 50 μM of CoPP for 24 h, prior to harvesting to detect total HbCO levels via ELISA. PRV-infected PK-15 cells were treated with 50 μM of CoPP, in the presence or absence of 50 μg/ml of Hb. After 24 h, cells were harvested to assess PRV gB mRNA and protein expression levels via **(E)** RT-qPCR and **(F)** western blotting, respectively, while **(G)** supernatants were harvested to measure virus copies via RT-qPCR. PRV-infected PK-15 or ST cells were incubated with 3% FBS + DMEM containing 0, 10, 25, and 50 μM of CORM-2 or 50 μM of inactive CORM-2 for 36 h. Cells and supernatants were collected for PRV replication analysis via **(I,K)** western blotting and **(H,J)** RT-qPCR. Data are presented as the mean ± standard deviation of three independent experiments. *P < 0.05, **P < 0.01, ***P < 0.001. CO, carbon monoxide; PRV, pseudorabies virus; HO-1, heme oxygenase-1; Hb, hemoglobin; ELISA, enzyme-linked immunosorbent assay; CoPP, cobalt-protoporphyrin; RT-qPCR, reverse transcription-quantitative PCR; CORM, CO-releasing molecules.

HO-1 yields three enzymatic downstream metabolites, including CO, which was assessed to determine whether it plays a role in the antiviral activity of HO-1 (Blancou et al., 2011). The intracellular content of CO was assessed following infection of PK-15 and ST cells with PRV. ELISA analysis demonstrated that PRV infection markedly decreased intracellular CO content in both PK-15 and ST cells (Figures 7A,B). Subsequently, intracellular CO content was assessed following HO-1 induction via CoPP. PK-15 cells were incubated with 50 μg/ml of Hb, in the presence or absence of 50 μM of CoPP, supernatants were collected after 36 h and HbCO levels were assessed via ELISA. The results demonstrated that HO-1 induction via CoPP notably increased supernatants HbCO content compared with the control group (Figures 7C,D), indicating that upregulation of HO-1 can promote endogenous CO production in host cells, although the quantity is small.

Overall, the results indicate that HO-1 may suppress PRV replication; however, the molecular mechanism underlying HO-1 inhibition of PRV replication still remains unclear. In order to investigate its antiviral molecular mechanism, PRV-infected PK-15 cells were treated with 50 μM of CoPP in the presence or absence of 50 μg/ml of Hb, or treated only by Hb. Cells and culture supernatants were harvested for detection of PRV gB mRNA and protein expression, and supernatant virus copies at 36 hpi. The results demonstrated that treatment with Hb partially reversed the inhibitory effect of CoPP on PRV gB mRNA (Figure 7E) and protein expression (Figure 7F). Furthermore, treatment with Hb in the presence of CoPP increased supernatant progeny virus copies compared with CoPP treatment alone (Figure 7G). However, treatment with Hb alone had no significant effect on PRV infection (Figures 7E–G). Taken together, these results indicate that endogenous CO produced by HO-1 catalysis partially mediates the antiviral effect of HO-1.

As an effective carrier of exogenous CO, CORM-2 was subsequently assessed to further determine the effect exogenous CO on PRV replication. PK-15 or ST cells were infected with PRV (0.01 MOI), followed by treatment with 0, 10, 25, 50 μM of CORM-2 or 50 μM of iCORM-2. Western blotting analysis demonstrated that CORM-2 suppressed PRV gB protein expression in both PK-15 (Figure 7H) and ST (Figure 7J) cells in a concentration-dependent manner, without inducing cytotoxic effects as observed with CoPP (data not shown). Conversely, iCORM-2 failed to exhibit a significant effect on gB expression (Figures 7H–J). Consistent with intracellular protein expression, CORM-2 was demonstrated to decrease supernatant virus copies in a concentration-dependent manner (Figures 7I–K); however, opposing effects were observed with iCORM-2. Taken together, these results suggest that CO partially mediates the antiviral activity of HO-1.

### HO-1 Metabolite, BV Partially Mediates the Inhibitory Effect of HO-1 on PRV Replication

**FIGURE 8:**
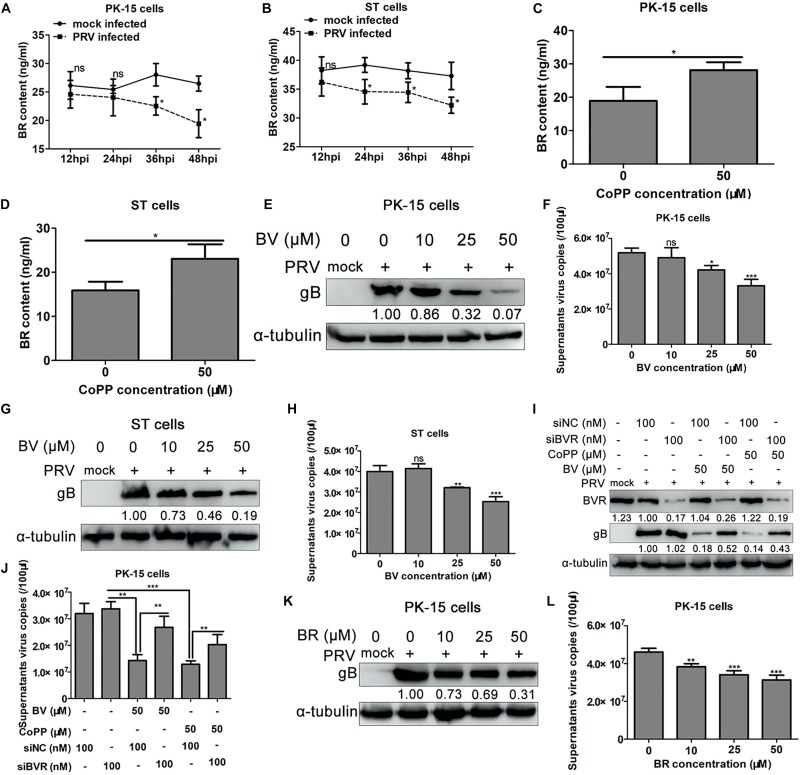
BV mediates the anti-PRV activity of HO-1. PK-15 **(A)** or ST **(B)** cells were infected with 0.01 MOI of PRV. Samples were collected at 12, 24, 36, 48 hpi for BR detection using ELISA. PK-15 **(C)** or ST **(D)** cells were treated with 50 μM CoPP for 24 h, the samples were used for BR content detection by ELISA. PK-15 or ST cells were infected with PRV (0.01 MOI) followed by treating with 0, 10, 25, 50 μM of BV for 36 h. Cells were harvested for gB detection using western blotting **(E,G)**, and supernatants were harvested for progeny virus copies measurement using RT-qPCR **(F,H)**. PK-15 cells were transfected with 100 nM of siBVR or siNC, then cells were inoculated with 0.01 MOI of PRV followed by treatment with 50 μM BV or CoPP. At 24 hpi, cells and supernatants were harvested, PRV gB was detected using western blotting **(I)**, progeny virus copies was detected using RT-qPCR **(J)**, respectively. **(K,L)** PRV-infected PK-15 cells were treated with 0, 10, 25, 50 μM of BR for 36 h. Cells and supernatants were collected for PRV replication analysis. Data are presented as the mean ± standard deviation of three independent experiments. *P < 0.05, **P < 0.01, ***P < 0.001. PRV, pseudorabies virus; BR, bilirubin; BV, biliverdin; ELISA, enzyme-linked immunosorbent assay; CoPP, cobalt-protoporphyrin; RT-qPCR, reverse transcription-quantitative PCR; MOI, multiplicity of infection.

Previous studies have demonstrated the antiviral effect of the HO-1 metabolite, BV (Lehmann et al., 2010; Zhu et al., 2010; Santangelo et al., 2012; Liu et al., 2016). In order to determine the role of BV in mediating the anti-PRV activity of HO-1, intracellular content of BR, the metabolite of BV, was assessed following PRV infection or treatment with CoPP via ELISA. Progression of PRV infection was associated with a downward trend of BR content in both PK-15 and ST cells compared with the control group (Figures 8A,B). Conversely, treatment with CoPP increased intracellular BR levels in both PK-15 and ST cells compared with the untreated control group (Figures 8C,D). Whether treatment with CoPP increased intracellular BR content by stimulating BVR expression was subsequently investigated. The results demonstrated no significant changes of BVR mRNA and protein expression levels in both PK-15 and ST cells (Supplementary Figure 4). Since the BV-BVR signaling cascade is the only signaling pathway that generates BR (Wegiel et al., 2011), it was assumed that increased BR content was directly due to HO-1 induction. In order to determine whether BV partially mediated the anti-PRV activity of HO-1, PRV-infected PK-15 or ST cells were incubated with 0, 10, 25, 50 μM of BV for 36 h at 37°C, and samples were subsequently harvested for viral replication analysis. A CCK-8 assay was subsequently performed to determine the cytotoxic effect of BV. Treatment with BV notably decreased PRV gB protein expression in PK-15 or ST cells (Figures 8E,G), as well as supernatant virus copies (Figures 8F,H), in a dose-dependent manner. However, no significant cytotoxic effect of BV was observed within the concentrations used in the present study (Supplementary Figures 5A,B). Taken together, these results suggest that like CO, BV also partially mediates the inhibitory effect of HO-1 on PRV replication.

In mammalian cells, BV is rapidly converted to BR via BVR catalysis (Wegiel et al., 2011). In order to determine whether BR also exerts anti-PRV effects, PK-15 cells were transfected with BVR-specific siRNA to prevent the conversion of BV to BR, which resulted in the accumulation of BV in PK-15 cells. PK-15 cells transfected with siBVR or siNC were subsequently infected with PRV (0.01 MOI), followed by treatment with or without 50 μM of BV or CoPP. Cells and culture supernatants were harvested at 36 hpi to detect PRV gB protein expression and progeny virus copies. Western blot analysis demonstrated no significant difference in PRV gB protein expression between PK-15 cells transfected with siBVR or siNC, without BV or CoPP treatment (Figure 8I). Conversely, transfection with siBVR, followed by BV treatment indicated increased PRV gB expression compared with siNC-transfected cells (Figure 8I). Besides, transfection with siBVR, followed by CoPP treatment indicated increased PRV gB expression compared with siNC-transfected cells as well (Figure 8I). Similar results were observed with regards to supernatant virus copies under the same experimental conditions (Figure 8J). Subsequently, exogenous BR was used to treat PRV-infected PK-15 cells and viral replication was determined, in order to further validate the aforementioned results. BR restrained both gB expression (Figure 8K) and supernatant virus copies (Figure 8L), in a concentration-dependent manner, while no significant cytotoxic effect was observed according to the results of the CCK-8 assay (Supplementary Figures 5C,D). Taken together, these results suggest that the secondary metabolite of BV, BR, contributes to the anti-PRV effect of HO-1.

## Discussion

PRV, a member of the α herpesvirus family, is considered infectious to most mammals, which highlights the necessity to develop antiviral strategies as a complementary approach to vaccination. Induction of HO-1 can protect organs or tissues from several types of stresses, including inflammation, oxidative injury and hemorrhage-induced hypoxia (Hashiba et al., 2001; Vallabhaneni et al., 2010). Furthermore, recent studies have reported the antiviral properties of HO-1 and its enzymatic products, emphasizing the key role HO-1 plays in protecting the host against viral infection. However, the molecular mechanism underlying HO-1 function in PRV infection and replication still remains unclear. To the best of our knowledge, the present study was the first to demonstrate that upregulated HO-1 expression inhibits PRV replication in both PK-15 and ST cells, indicating the antiviral potential of this cytoprotective enzyme during PRV infection. Furthermore, the results demonstrated that the downstream metabolites of HO-1, BV and CO, mediate its antiviral effects, elucidating the molecular mechanism by which HO-1 inhibits viral replication.

Previous studies indicated that both HBV and HCV infection caused a down-regulation of HO-1 as a means of optimizing viral replication (Abdalla et al., 2004; Protzer et al., 2007). Consistent with previous finding, the results of the present study indicated that PRV infection was associated with decreased HO-1 expression in both PK-15 and ST cells (Figure 1). Thus it was speculated that the virus develops multiple strategies to evade host antiviral defense responses, by which suppression of HO-1 expression is a manifestation of this strategy. Some previous studies have certificated that HO-1 expression is regulated by transcription factors, including Nrf2, Keap1 and Bach1, which bind to the antioxidant response element in the promoter region of HO-1 (Han, 2011; Fredenburgh et al., 2015). However, although PRV replication is necessary for HO-1 inhibition in the present experimental model, it still remains unclear as to whether viral proteins are involved in suppressing activation of the aforementioned transcription factors, thus abrogating HO-1 expression. Whether viral protein directly interacts with HO-1 requires further investigation, and possesses important implications to understanding the molecular mechanism underlying HO-1 downregulation. Some evidence suggests that host cell oxidative stress status plays an important role in regulating viral replication and infectivity (Cai et al., 2003). Up-regulation of intracellular oxidative stress and down-regulation of HO-1 in PRV-infected cells may indicate that PRV can induce oxidative stress in host cells and antagonizes the HO-1 pathway to facilite self-replication. Certain porphyrins and porphyrin derivatives possess virucidal effect against some other viruses, such as EBOV, HIV and MARV (Vzorov et al., 2002; Chen-Collins et al., 2003; Guo et al., 2011), thus the potential of virucidal effect of CoPP was evaluated. Co-incubation of PRV with CoPP had obvious, but not complete inhibitory effect on PRV proliferative property (Figures 6G,H), demonstrating certain virucidal activity. The molecular mechanism underlying how porphyrins such as CoPP disrupt the PRV enveloped particles is unknown. However, the evidence that CoPP does not affect viral attachment or internalization into host cells suggest that CoPP directly disrupts the virus as a virucidal agent which may interact with specific structures (glycosylation, phosphorylation, peptide interface, etc.) on the viral particle surface or lipids within the viral envelope, as some studies have suggested that porphyrins abrogate infection by HIV-1 and that this activity appears to be a result of an interaction with the envelope protein. Since all the viral envelopes have a lipid bilayer basal structure similar to that of the cell membrane (Seeger and Mason, 2000) and these compounds are well tolerated by cells, it is unlikely that porphyrin compounds disrupt envelope through lipid depletion. Besides, porphyrins were reported to selectively inactivated a diversity of enveloped viruses, but not non-enveloped viruses (Guo et al., 2011), implies that some common structural features presented on the surfaces of viral envelope proteins are the most possible targets.

In order to verify the specificity of upregulated HO-1 in mediating the antiviral effect of CoPP, HO-1-specific siRNA was used to downregulate CoPP-induced HO-1 expression. Downregulation of HO-1 partially reversed the CoPP-induced inhibitory effect on PRV replication, proving that HO-1 specifically mediates the antiviral activity of CoPP. Overexpression of HO-1 suppressed PRV replication, while knockdown of basal levels of HO-1 promoted PRV replication in both PK-15 and ST cells, indicating that aberrant HO-1 expression may effectively inhibit PRV replication. ZnPP, a HO-1 specific inhibitor that can upregulate HO-1 expression, while inhibiting its enzymatic activity was used to determine whether HO-1 activity was essential to inhibit PRV replication (Sardana and Kappas, 1987). Treatment with ZnPP partially reversed CoPP-induced PRV inhibition, but failed to attenuate PRV replication, suggesting that HO-1 activity is essential for PRV attenuation. The present study confirmed the anti-PRV activity of HO-1; however, this property does not seem to be ubiquitous. For example, HO-1 has been reported to promote KSHV replication in endothelial cells via its downstream metabolite, CO (Botto et al., 2017). Furthermore, HO-1 failed to play a suppressive role in the replication of VSV (Hill-Batorski et al., 2013), indicating different physiological functions of HO-1 against different pathogenic microorganisms. It is hypothesized that these inconsistencies may attribute to the specific molecular mechanisms underlying the antiviral properties of HO-1.

The roles of HO-1 downstream metabolites were subsequently investigated to determine the precise molecular mechanisms underlying the inhibition of PRV replication by HO-1. Both BV and CO mediated the antiviral activity of HO-1, as demonstrated by inhibition of PRV replication via BV or CORM-2 treatment in cell-based assays. As a downstream metabolite of HO-1, BV has been reported to exert antiviral activity against several domestic animal viruses, including BVDV (Ma et al., 2017). Considering that the majority of organs retain BVR activity to transform BV into BR (Maines, 2005), and the BV-BVR signaling pathway is the only source of intracellular BR (Maines, 2005), the present study set out to determine whether the antiviral activity of BV against PRV attributed to its conversion into BR rather than a direct effect of BV, in PK-15 and ST cells. Knockdown of BVR exerted no significant effect on PRV replication, whereas co-treatment with siBVR and BV or CoPP partially decreased inhibition of PRV replication compared with the siNC and BV or CoPP co-treatment group, suggesting that the conversion of BV to BR may participate in the antiviral effect of BV. As expected, treatment with BR notably suppressed PRV replication in PK-15 cells, further manifesting its antiviral activity. A previous study reported that BR exerts antiviral activity against HSV-1 infection via NO production (Santangelo et al., 2012), and NO synthesized by NOS has been demonstrated to enhance the activity of HO-1, resulting in an activating positive loop between NO and CO (Zuckerbraun et al., 2003; Wegiel et al., 2010). Thus, the antiviral effect of BV/BR against PRV may be due to the synergistic effect between NO and CO; however, this requires further investigation.

HO-1 induction and endogenous CO have been demonstrated to take on a protective role in some animal disease models and *in vitro* experiments (Otterbein et al., 2003; Knauert et al., 2013). Furthermore, the HO-1/CO axis has favorable properties against inflammatory responses (Chung et al., 2009). The present study demonstrated that the protective effect of HO-1 against PRV may be achieved via administration of CORM-2, but not iCORM-2, suggesting that besides BV, CO may also exert cytoprotective effects during PRV infection. HO-1-derived CO was also indicated to play a key role in the antiviral activity of HO-1, whereby CO scavenger Hb partially reversed the inhibitory effect of CoPP-induced HO-1 on PRV replication. Previous studies have demonstrated that CO decreases the replication ability of PRRSV by suppressing the activation of the NF-κB signaling pathway, which plays a significant role in PRV replication (Zhang et al., 2017; Zhao et al., 2017). Thus, the NF-κB signaling pathway may act as a potential target for the HO-1/CO system to exert its antiviral effect. According to previous reports, HO-1 and its metabolite, CO, function as important immune regulatory factors (Remy et al., 2009; Blancou et al., 2011), thus, the HO-1/CO system may be used as an antiviral or innate immune mechanism by host cells infected with PRV. Furthermore, HO-1/CO have been reported to directly inhibit heat shock protein (HSP) 90 activity of host cells, which plays a critical role in nuclear transport of HSV-1 capsid protein (Lee W.Y. et al., 2014; Zhong et al., 2014). Considering that both PRV and HSV-1 belong to the α Herpesviridae family and have similar biological characteristics, CO may interfere with HSP90 activity to suppress PRV replication in host cells; however, this requires further investigation. Previous studies have also reported a synergistic effect between BV and CO when the host is faced with noxious stimuli or infected with certain viruses. For example, BV and CO have been demonstrated to exhibit additive effects in protecting the liver from immune-mediated injury or Huh7.5 cells from HBV infection (Sass et al., 2004; Lehmann et al., 2010). The antiviral effect of HO-1 against PRV may be achieved by administrating either BV or CO, whereby the synergistic effect of the two downstream metabolites is hypothesized to mediate the antiviral effect of HO-1. Despite the novel insights, the present study was unable to determine the downstream molecular mechanisms underlying the antiviral effects of BV/BR and CO; thus, prospective studies will focus on these associations.

Taken together, the results of the present study suggest that HO-1 exerts an antiviral effect against PRV, and these functions are at least partially mediated by its downstream products, BV and CO. These findings provide novel insight into the molecular mechanisms underlying HO-1 inhibition on PRV replication, while also indicating potential novel measures for controlling PRV infection.

## Data Availability Statement

All datasets generated for this study are included in the article/Supplementary Material.

## Author Contributions

AZ, GZ, and YD conceived and designed the experiments. AZ and BW performed the experiments. YW, DJ, and PJ analyzed the data. AZ and GZ wrote the manuscript.

## Conflict of Interest

The authors declare that the research was conducted in the absence of any commercial or financial relationships that could be construed as a potential conflict of interest.

## References

[B1] Abdalla M. Y. Britigan B. E. Wen F. Icardi M. McCormick M. L. LaBrecque D. R. (2004). Down-regulation of heme oxygenase-1 by hepatitis C virus infection in vivo and by the in vitro expression of hepatitis C core protein. *J. Infect. Dis.* 190 1109–1118. 10.1086/423488 15319861

[B2] An T. Q. Peng J. M. Tian Z. J. Zhao H. Y. Li N. Liu Y. M. (2013). Pseudorabies virus variant in Bartha-K61-vaccinated pigs. China, 2012. *Emerg. Infect.Dis.* 19 1749–1755. 10.3201/eid1911.130177 24188614 PMC3837674

[B3] Bach F. H. (2006). Heme oxygenase-1 and transplantation tolerance. *Hum. Immunol.* 67 430–432. 10.1016/j.humimm.2006.03.006 16728265

[B4] Blancou P. Tardif V. Simon T. Remy S. Carreno L. Kalergis A. (2011). Immunoregulatory properties of heme oxygenase-1. *Methods Mol.Biol.* 677 247–268. 10.1007/978-1-60761-869-0_18 20941616

[B5] Botto S. Gustin J. K. Moses A. V. (2017). The heme metabolite carbon monoxide facilitates KSHV Infection by inhibiting TLR4 signaling in endothelial cells. *Front. Microbiol.* 8:568. 10.3389/fmicb.2017.00568 28421060 PMC5376558

[B6] Cai J. Chen Y. Seth S. Furukawa S. Compans R. W. Jones D. P. (2003). Inhibition of influenza infection by glutathione. *Free Radic. Biol. Med.* 34 928–936. 10.1016/s0891-5849(03)00023-6 12654482

[B7] Chen-Collins A. R. Dixon D. W. Vzorov A. N. Marzilli L. G. Compans R. W. (2003). Prevention of poxvirus infection by tetrapyrroles. *BMC Infect. Dis.* 3:9. 10.1186/1471-2334-3-9 12773208 PMC166128

[B8] Cheshenko N. Trepanier J. B. Stefanidou M. Buckley N. Gonzalez P. Jacobs W. (2013). HSV activates Akt to trigger calcium release and promote viral entry: novel candidate target for treatment and suppression. *FASEB J.* 27 2584–2599. 10.1096/fj.12-220285 23507869 PMC3688744

[B9] Choi B. M. Pae H. O. Jeong Y. R. Oh G. S. Jun C. D. Kim B. R. (2004). Overexpression of heme oxygenase (HO)-1 renders Jurkat T cells resistant to fas-mediated apoptosis: involvement of iron released by HO-1. *Free Radic. Bio. Med.* 36 858–871. 10.1016/j.freeradbiomed.2004.01.004 15019971

[B10] Chung S. W. Hall S. R. Perrella M. A. (2009). Role of haem oxygenase-1 in microbial host defence. *Cell. Microbiol.* 11 199–207. 10.1111/j.1462-5822.2008.01261.x 19016784 PMC3080039

[B11] Chung S. W. Liu X. Macias A. A. Baron R. M. Perrella M. A. (2008). Heme oxygenase-1-derived carbon monoxide enhances the host defense response to microbial sepsis in mice. *J. Clin. Investig.* 118 239–247. 10.1172/JCI32730 18060048 PMC2104480

[B12] Devadas K. Dhawan S. (2006). Hemin activation ameliorates HIV-1 infection via heme oxygenase-1 induction. *J. Immunol.* 176 4252–4257. 10.4049/jimmunol.176.7.4252 16547262

[B13] Eisenstein R. S. Garcia-Mayol D. Pettingell W. Munro H. N. (1991). Regulation of ferritin and heme oxygenase synthesis in rat fibroblasts by different forms of iron. *Proc. Natl. Acad. Sci. U.S.A.* 88 688–692. 10.1073/pnas.88.3.688 1992460 PMC50878

[B14] Espinoza J. A. Leon M. A. Cespedes P. F. Gomez R. S. Canedo-Marroquin G. Riquelme S. A. (2017). Heme Oxygenase-1 modulates human respiratory syncytial virus replication and lung pathogenesis during infection. *J. Immunol.* 199 212–223. 10.4049/jimmunol.1601414 28566367

[B15] Fillebeen C. Rivas-Estilla A. M. Bisaillon M. Ponka P. Muckenthaler M. Hentze M. W. (2005). Iron inactivates the RNA polymerase NS5B and suppresses subgenomic replication of hepatitis C Virus. *J. Biol.Chem.* 280 9049–9057. 10.1074/jbc.M412687200 15637067

[B16] Fredenburgh L. E. Merz A. A. Cheng S. (2015). Haeme oxygenase signalling pathway: implications for cardiovascular disease. *Eur. Heart J.* 36 1512–1518. 10.1093/eurheartj/ehv114 25827602 PMC4475572

[B17] Freuling C. M. Muller T. F. Mettenleiter T. C. (2017). Vaccines against pseudorabies virus (PrV). *Vet. Microbiol.* 206 3–9. 10.1016/j.vetmic.2016.11.019 27890448

[B18] Greenberg D. A. (2002). The jaundice of the cell. *Proc. Nat.l. Acad. Sci.U. S.A.* 99 15837–15839. 10.1073/pnas.012685199 12461187 PMC138521

[B19] Guo H. Pan X. Mao R. Zhang X. Wang L. Lu X. (2011). Alkylated porphyrins have broad antiviral activity against hepadnaviruses, flaviviruses, filoviruses, and arenaviruses. *Antimicrob. Agents Chemother.* 55 478–486. 10.1128/AAC.00989-10 21135183 PMC3028764

[B20] Han O. (2011). Molecular mechanism of intestinal iron absorption. *Metallomics* 3 103–109. 10.1039/c0mt00043d 21210059

[B21] Hashiba T. Suzuki M. Nagashima Y. Suzuki S. Inoue S. Tsuburai T. (2001). Adenovirus-mediated transfer of heme oxygenase-1 cDNA attenuates severe lung injury induced by the influenza virus in mice. *Gene Ther.* 8 1499–1507. 10.1038/sj.gt.3301540 11593363

[B22] Hill-Batorski L. Halfmann P. Neumann G. Kawaoka Y. (2013). The cytoprotective enzyme heme oxygenase-1 suppresses Ebola virus replication. *J. Virol.* 87 13795–13802. 10.1128/JVI.02422-13 24109237 PMC3838215

[B23] Kapitulnik J. Maines M. D. (2009). Pleiotropic functions of biliverdin reductase: cellular signaling and generation of cytoprotective and cytotoxic bilirubin. *Trends Pharmacol. Sci.* 30 129–137. 10.1016/j.tips.2008.12.003 19217170

[B24] Knauert M. Vangala S. Haslip M. Lee P. J. (2013). Therapeutic applications of carbon monoxide. *Oxid. Med. Cell. Longev.* 2013:360815. 10.1155/2013/360815 24648866 PMC3932177

[B25] Lee J. C. Tseng C. K. Young K. C. Sun H. Y. Wang S. W. Chen W. C. (2014). Andrographolide exerts anti-hepatitis C virus activity by up-regulating haeme oxygenase-1 via the p38 MAPK/Nrf2 pathway in human hepatoma cells. *Br. J. Pharmacol.* 171 237–252. 10.1111/bph.12440 24117426 PMC3874710

[B26] Lee W. Y. Chen Y. C. Shih C. M. Lin C. M. Cheng C. H. Chen K. C. (2014). The induction of heme oxygenase-1 suppresses heat shock protein 90 and the proliferation of human breast cancer cells through its byproduct carbon monoxide. *Toxicol. Appl. Pharmacol.* 274 55–62. 10.1016/j.taap.2013.10.027 24211270

[B27] Lehmann E. El-Tantawy W. H. Ocker M. Bartenschlager R. Lohmann V. Hashemolhosseini S. (2010). The heme oxygenase 1 product biliverdin interferes with hepatitis C virus replication by increasing antiviral interferon response. *Hepatology* 51 398–404. 10.1002/hep.23339 20044809

[B28] Liu X. M. Durante Z. E. Peyton K. J. Durante W. (2016). Heme oxygenase-1-derived bilirubin counteracts HIV protease inhibitor-mediated endothelial cell dysfunction. *Free Radic. Biol. Med.* 94 218–229. 10.1016/j.freeradbiomed.2016.03.003 26968795 PMC4844824

[B29] Ma Z. Pu F. Zhang X. Yan Y. Zhao L. Zhang A. (2017). Carbon monoxide and biliverdin suppress bovine viral diarrhoea virus replication. *J. Gen. Virol.* 98 2982–2992. 10.1099/jgv.0.000955 29087274

[B30] Mackern-Oberti J. P. Obreque J. Mendez G. P. Llanos C. Kalergis A. M. (2015). Carbon monoxide inhibits T cell activation in target organs during systemic lupus erythematosus. *Clini. Exp. Immunol.* 182 1–13. 10.1111/cei.12657 26095291 PMC4578503

[B31] Maines M. D. (2005). New insights into biliverdin reductase functions: linking heme metabolism to cell signaling. *Physiology* 20 382–389. 10.1152/physiol.00029.2005 16287987

[B32] Mettenleiter T. C. (1991). Molecular biology of pseudorabies (Aujeszky’s disease) virus. *Comp. Immunol. Microbiol. Infect. Dis.* 14 151–163. 10.1016/0147-9571(91)90128-z 1657509

[B33] Mettenleiter T. C. (2000). Aujeszky’s disease (pseudorabies) virus: the virus and molecular pathogenesis–state of the art. June 1999. *Vet. Res.* 31 99–115. 10.1051/vetres:2000110 10726640

[B34] Motterlini R. Clark J. E. Foresti R. Sarathchandra P. Mann B. E. Green C. J. (2002). Carbon monoxide-releasing molecules: characterization of biochemical and vascular activities. *Circ. Res.* 90 E17–E24. 11834719 10.1161/hh0202.104530

[B35] Nauwynck H. Glorieux S. Favoreel H. Pensaert M. (2007). Cell biological and molecular characteristics of pseudorabies virus infections in cell cultures and in pigs with emphasis on the respiratory tract. *Vet. Res.* 38 229–241. 10.1051/vetres:200661 17257571

[B36] Olagnier D. Peri S. Steel C. van Montfoort N. Chiang C. Beljanski V. (2014). Cellular oxidative stress response controls the antiviral and apoptotic programs in dengue virus-infected dendritic cells. *PLoS Pathog.* 10:e1004566. 10.1371/journal.ppat.1004566 25521078 PMC4270780

[B37] Otterbein L. E. (2002). Carbon monoxide: innovative anti-inflammatory properties of an age-old gas molecule. *Antioxid. Redox Signal.* 4 309–319. 10.1089/152308602753666361 12006182

[B38] Otterbein L. E. Zuckerbraun B. S. Haga M. Liu F. Song R. Usheva A. (2003). Carbon monoxide suppresses arteriosclerotic lesions associated with chronic graft rejection and with balloon injury. *Nat. Med.* 9 183–190. 10.1038/nm817 12539038

[B39] Protzer U. Seyfried S. Quasdorff M. Sass G. Svorcova M. Webb D. (2007). Antiviral activity and hepatoprotection by heme oxygenase-1 in hepatitis B virus infection. *Gastroenterology* 133 1156–1165. 10.1053/j.gastro.2007.07.021 17919491

[B40] Remy S. Blancou P. Tesson L. Tardif V. Brion R. Royer P. J. (2009). Carbon monoxide inhibits TLR-induced dendritic cell immunogenicity. *J. Immunol.* 182 1877–1884. 10.4049/jimmunol.0802436 19201840

[B41] Riquelme S. A. Bueno S. M. Kalergis A. M. (2015a). Carbon monoxide down-modulates Toll-like receptor 4/MD2 expression on innate immune cells and reduces endotoxic shock susceptibility. *Immunology* 144 321–332. 10.1111/imm.12375 25179131 PMC4298426

[B42] Riquelme S. A. Pogu J. Anegon I. Bueno S. M. Kalergis A. M. (2015b). Carbon monoxide impairs mitochondria-dependent endosomal maturation and antigen presentation in dendritic cells. *Eur. J. Immunol.* 45 3269–3288. 10.1002/eji.201545671 26461179

[B43] Ryter S. W. Choi A. M. (2009). Heme oxygenase-1/carbon monoxide: from metabolism to molecular therapy. *Ame. J. Respir. Cell Mol. Biol.* 41 251–260. 10.1165/rcmb.2009-0170TR 19617398 PMC2742746

[B44] Ryter S. W. Choi A. M. (2016). Targeting heme oxygenase-1 and carbon monoxide for therapeutic modulation of inflammation. *Transl. Res.* 167 7–34. 10.1016/j.trsl.2015.06.011 26166253 PMC4857893

[B45] Santangelo R. Mancuso C. Marchetti S. Di Stasio E. Pani G. Fadda G. (2012). Bilirubin: an endogenous molecule with antiviral activity in vitro. *Front. Pharmacol.* 3:36. 10.3389/fphar.2012.00036 22408623 PMC3297833

[B46] Sardana M. K. Kappas A. (1987). Dual control mechanism for heme oxygenase: tin(IV)-protoporphyrin potently inhibits enzyme activity while markedly increasing content of enzyme protein in liver. *Proc. Natl. Acade. Sci. U.S.A.* 84 2464–2468. 10.1073/pnas.84.8.2464 3470805 PMC304672

[B47] Sass G. Seyfried S. Parreira Soares M. Yamashita K. Kaczmarek E. Neuhuber W. L. (2004). Cooperative effect of biliverdin and carbon monoxide on survival of mice in immune-mediated liver injury. *Hepatology* 40 1128–1135. 10.1002/hep.20450 15486963

[B48] Seeger C. Mason W. S. (2000). Hepatitis B virus biology. *Microbiol. Mo. Biol.Rev.* 64 51–68. 10.1128/mmbr.64.1.51-68.2000 10704474 PMC98986

[B49] Tenhunen R. Marver H. S. Schmid R. (1969). Microsomal heme oxygenase. Characterization of the enzyme. *J. Biol. Chem.* 244 6388–6394. 4390967

[B50] Tseng C. K. Lin C. K. Wu Y. H. Chen Y. H. Chen W. C. Young K. C. (2016). Human heme oxygenase 1 is a potential host cell factor against dengue virus replication. *Scie. Rep.* 6:32176. 10.1038/srep32176 27553177 PMC4995454

[B51] Tung W. H. Hsieh H. L. Lee I. T. Yang C. M. (2011). Enterovirus 71 induces integrin beta1/EGFR-Rac1-dependent oxidative stress in SK-N-SH cells: role of HO-1/CO in viral replication. *J. Cell.Physiol.* 226 3316–3329. 10.1002/jcp.22677 21321939

[B52] Vallabhaneni R. Kaczorowski D. J. Yaakovian M. D. Rao J. Zuckerbraun B. S. (2010). Heme oxygenase 1 protects against hepatic hypoxia and injury from hemorrhage via regulation of cellular respiration. *Shock* 33 274–281. 10.1097/SHK.0b013e3181b0f566 19536046

[B53] Vzorov A. N. Dixon D. W. Trommel J. S. Marzilli L. G. Compans R. W. (2002). Inactivation of human immunodeficiency virus type 1 by porphyrins. *Antimicrob. Agents Chemother.* 46 3917–3925. 10.1128/aac.46.12.3917-3925.2002 12435696 PMC132794

[B54] Wegiel B. Gallo D. Csizmadia E. Roger T. Kaczmarek E. Harris C. (2011). Biliverdin inhibits Toll-like receptor-4 (TLR4) expression through nitric oxide-dependent nuclear translocation of biliverdin reductase. *Proc. NatL. Acad. Sci. U.S.A.* 108 18849–18854. 10.1073/pnas.1108571108 22042868 PMC3219137

[B55] Wegiel B. Gallo D. J. Raman K. G. Karlsson J. M. Ozanich B. Chin B. Y. (2010). Nitric oxide-dependent bone marrow progenitor mobilization by carbon monoxide enhances endothelial repair after vascular injury. *Circulation* 121 537–548. 10.1161/CIRCULATIONAHA.109.887695 20083679 PMC2841354

[B56] Xiao S. Zhang A. Zhang C. Ni H. Gao J. Wang C. (2014). Heme oxygenase-1 acts as an antiviral factor for porcine reproductive and respiratory syndrome virus infection and over-expression inhibits virus replication in vitro. *Antivir. Res.* 110 60–69. 10.1016/j.antiviral.2014.07.011 25086213

[B57] Yu X. Zhou Z. Hu D. Zhang Q. Han T. Li X. (2014). Pathogenic pseudorabies virus. China, 2012. *Emerg. Infect. Dis.* 20 102–104. 10.3201/eid2001.130531 24377462 PMC3884716

[B58] Zhang A. Zhao L. Li N. Duan H. Liu H. Pu F. (2017). Carbon monoxide inhibits porcine reproductive and respiratory syndrome virus replication by the Cyclic GMP/Protein Kinase G and NF-kappaB signaling pathway. *J Virol.* 91:e01866-16. 10.1128/JVI.01866-16 27795439 PMC5165190

[B59] Zhao X. Cui Q. Fu Q. Song X. Jia R. Yang Y. (2017). Antiviral properties of resveratrol against pseudorabies virus are associated with the inhibition of IkappaB kinase activation. *Scie. Rep* 7:8782. 10.1038/s41598-017-09365-0 28821840 PMC5562710

[B60] Zhong M. Zheng K. Chen M. Xiang Y. Jin F. Ma K. (2014). Heat-shock protein 90 promotes nuclear transport of herpes simplex virus 1 capsid protein by interacting with acetylated tubulin. *PloS One* 9:e99425. 10.1371/journal.pone.0099425 24901434 PMC4047101

[B61] Zhu Z. Wilson A. T. Luxon B. A. Brown K. E. Mathahs M. M. Bandyopadhyay S. (2010). Biliverdin inhibits hepatitis C virus nonstructural 3/4A protease activity: mechanism for the antiviral effects of heme oxygenase? *Hepatology* 52 1897–1905. 10.1002/hep.23921 21105106 PMC3058505

[B62] Zuckerbraun B. S. Billiar T. R. Otterbein S. L. Kim P. K. Liu F. Choi A. M. (2003). Carbon monoxide protects against liver failure through nitric oxide-induced heme oxygenase 1. *J. Exp. Med.* 198 1707–1716. 10.1084/jem.20031003 14657222 PMC2194127

