# Supplementary material for: The Cytoprotective Enzyme Heme Oxygenase-1 Suppresses Pseudorabies Virus Replication in vitro
Source: Front Microbiol. 2020 Mar 13;11:412. doi: 10.3389/fmicb.2020.00412 (PMC7082841; doi:10.3389/fmicb.2020.00412)
Supplement: Supplementary file 1 [file Table_1.DOCX]

**Supplemental figures:**

**
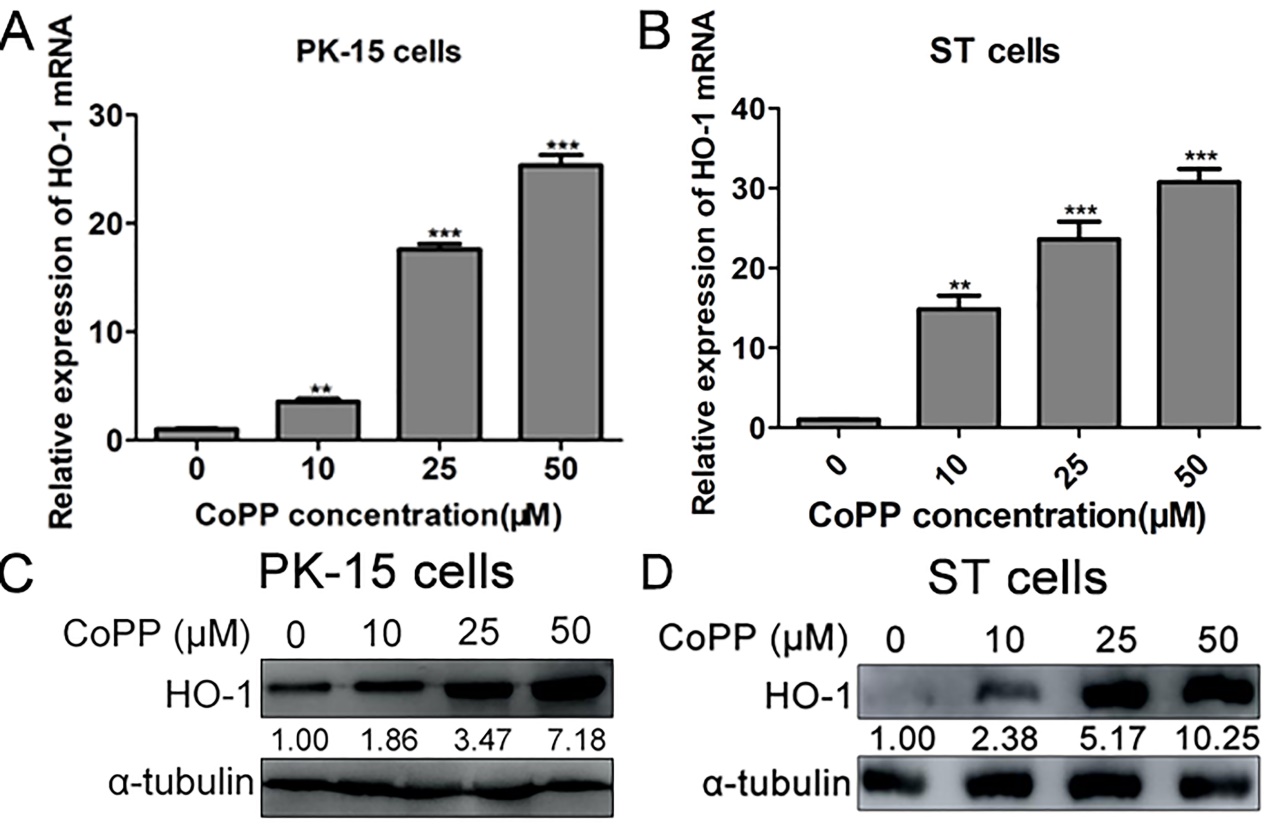
**

**FIGURE 1 CoPP concentration dependent up-regulates HO-1 expression in PK-15 and ST cells.** PK-15 (A and C) or ST cells (B and D) were treated with 0, 10, 25, 50 μM of CoPP for 24 h, then cells were harvested for detection of HO-1 mRNA expression using RT-qPCR, and for detection of HO-1 protein expression using western blotting. ^**^P<0.01, ^***^P<0.001. CoPP, cobalt-protoporphyrin; RT-qPCR, reverse transcription-quantitative PCR; HO-1, heme oxygenase-1.


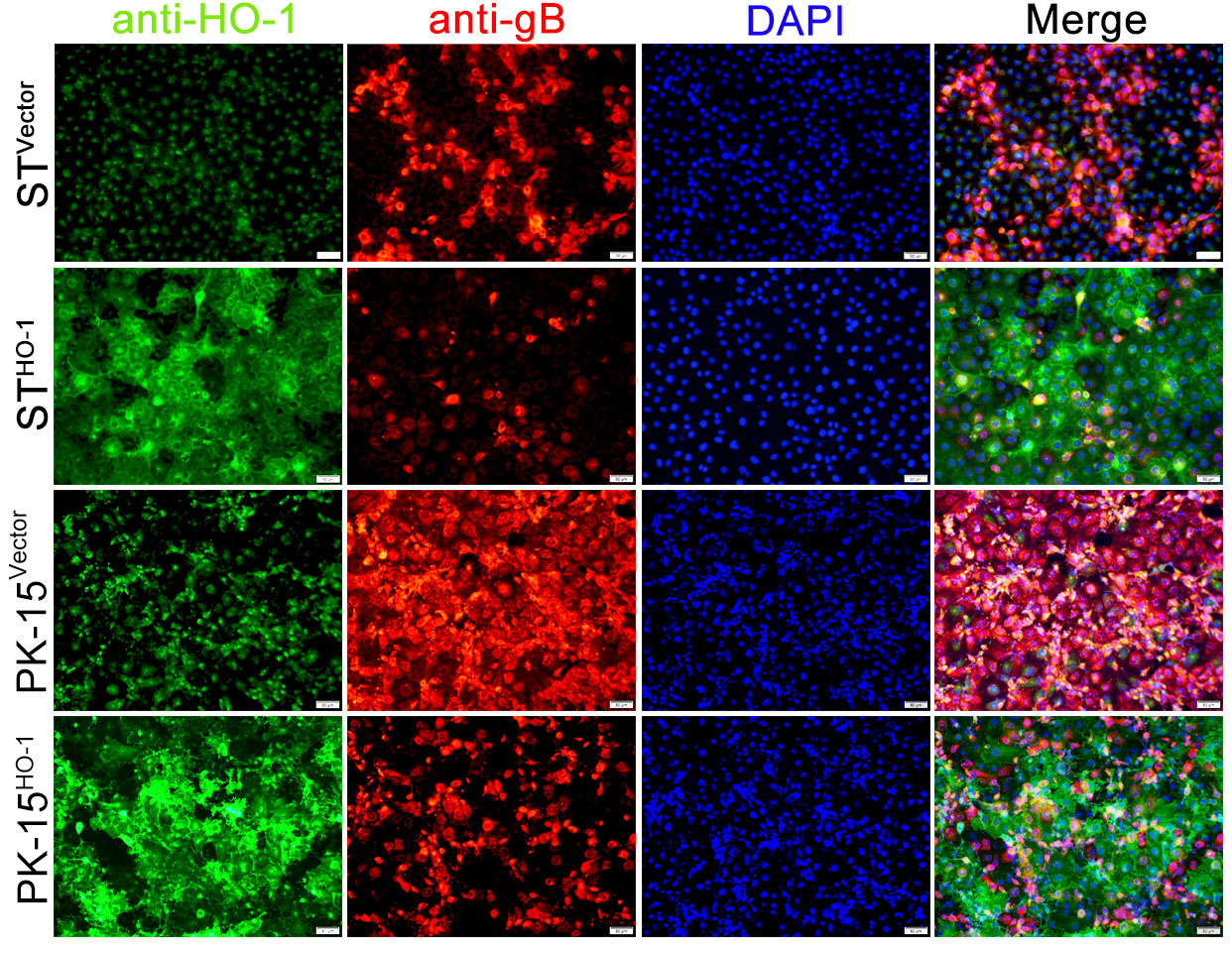


**FIGURE 2 Overexpression of HO-1 suppresses PRV infection of PK-15 and ST cells.** PK-15^Vector^ and PK-15^HO-1^ or ST^Vector^ and ST^HO-1^ cells were infected with 0.01 MOI of PRV for 1 h at 37^o^C, then cells were fixed with -20^o^C pre-cooled 70% ethanol at 24 hpi. IFA was conducted using mouse anti-HO-1 monoclonal antibody and rabbit anti-PRV gB polyclonal antibody, followed by incubating with Alexa 594-conjugated goat anti-mouse IgG H&L and Alexa 488-conjugated goat anti-rabbit IgG H&L, respectively. Images were representative of observations from three independent experiments (100 x).


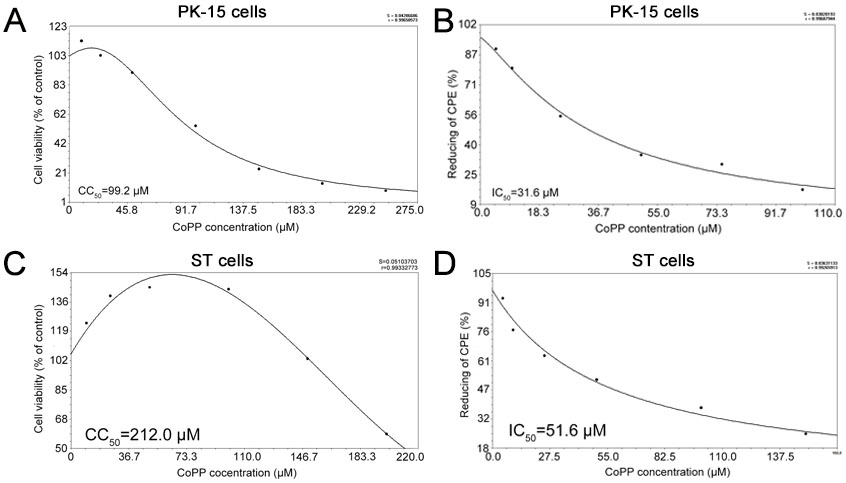


**FIGURE 3 The CC_50_ and IC_50_ values of CoPP in PK-15 and ST cells.**


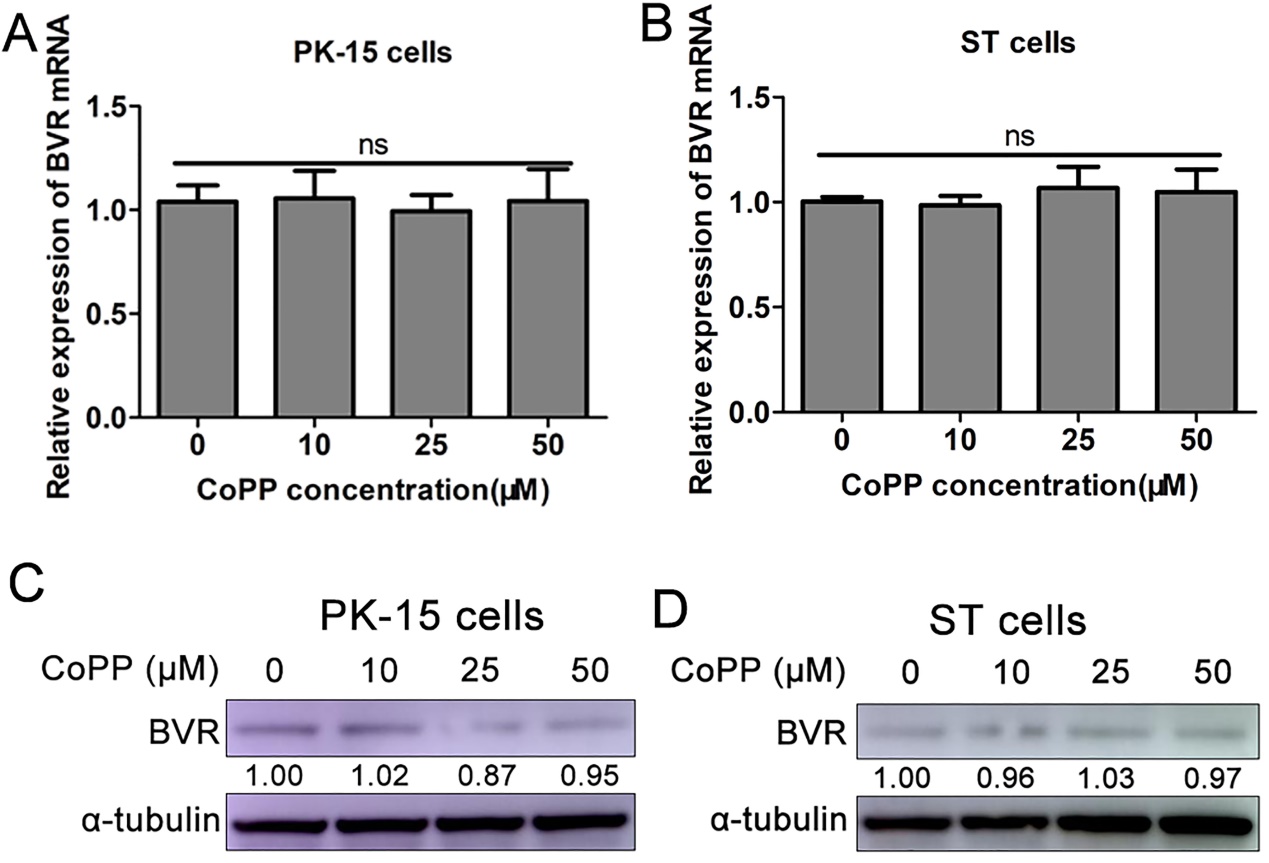


**FIGURE 4** PK-15 (A and C) or ST (B and D) cells were incubated with 0, 10, 25, 50 μM of CoPP. 24 h later, cells were harvested for BVR mRNA and protein detection by RT-qPCR and western blotting, respectively. CoPP, cobalt-protoporphyrin; RT-qPCR, reverse transcription-quantitative PCR; BVR, biliverdin reductase.


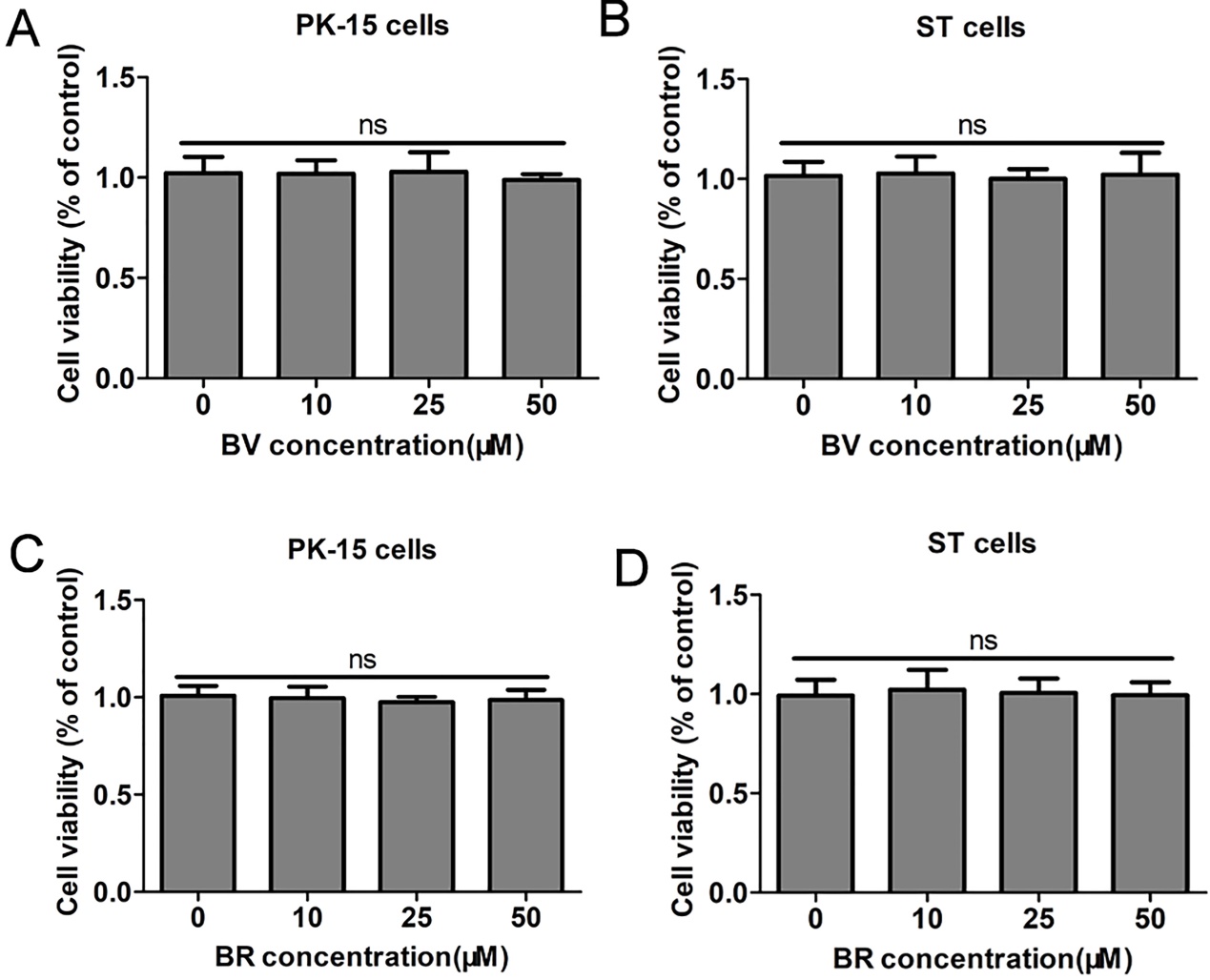


**FigS. 5 Biliverdin or bilirubin does not cause cytotoxic effect to PK-15 and ST cells.** PK-15 or ST cells were seeded into 96-well cell culture plate at a density of 1x 10^4^ cells/well. After culturing for 24 h at 37^o^C with 5% CO_2_, the old culture medium was replaced with 3% FBS+DMEM containing indicated concentrations of BV (A and B) or BR (C and D) and continued culturing for another 36 h. Then 10 μl CCK-8 reagent was added to each well of the 96-well plate containing 100 μl fresh DMEM medium and incubated for 2 h at 37^o^C. The absorbance of each well at 450 nM was measured, and cell viability was calculated based on the absorbance value.
